# PfATP2 is a flippase on the *Plasmodium falciparum* surface that is important for growth and influences parasite sensitivity to antiplasmodial compounds

**DOI:** 10.1371/journal.ppat.1013645

**Published:** 2025-10-27

**Authors:** Deyun Qiu, Eleanor England, Adele M. Lehane

**Affiliations:** Research School of Biology, Australian National University, Canberra, Australian Capital Territory, Australia; University of Geneva Faculty of Medicine: Universite de Geneve Faculte de Medecine, SWITZERLAND

## Abstract

Antimalarials play a crucial role in the fight against malaria. However, resistance of the most virulent malaria parasite, *Plasmodium falciparum*, to front-line antimalarials is spreading. To identify new antimalarials, millions of compounds have been screened for their ability to inhibit the growth of blood-stage *P. falciparum* parasites. To gain insight into the mode of action of novel compounds and the ease by which parasites can acquire resistance to them, many have been tested in ‘*in vitro* evolution’ experiments, in which parasites are exposed to the compound for a prolonged period of time. In a recent study, parasite resistance to two compounds, MMV007224 and MMV665852, was associated with amplification of the *pfatp2* gene, implicating PfATP2, a putative phospholipid flippase, as a parasite drug target or resistance determinant. These two compounds, along with MMV665794 (which is structurally related to MMV007224), had previously been reported to dysregulate pH in parasites. Here, we show that PfATP2 localises to the parasite surface and is important for parasite growth. We demonstrate that parasites genetically engineered to overexpress PfATP2 display reduced sensitivity to MMV665794, MMV007224 and MMV665852 compared to parasites with a normal expression level of the protein, and that parasites in which PfATP2 is knocked down become hypersensitive to the three compounds. We show that PfATP2 expression level does not affect the cytosolic pH of parasites, or the potency by which MMV665794 or MMV007224 dysregulate parasite pH. We show that PfATP2-overexpressing parasites internalise a fluorescent phosphatidylserine analogue (NBD-PS) at a greater rate than parasites with a normal expression level of PfATP2, and that parasites in which PfATP2 is knocked down have a reduced rate of NBD-PS uptake. Further, we provide evidence that MMV665794 and MMV007224 give rise to a reduction in NBD-PS internalisation. Together, our data are consistent with PfATP2 serving as a major ATP-dependent phosphatidylserine internalisation mechanism on the parasite plasma membrane, and being a potential target of MMV665794 and MMV007224.

## Author summary

Recent screens have identified numerous compounds active against the most virulent species of human malaria parasite, *Plasmodium falciparum*. Previously, researchers exposed parasite cultures to many such compounds, including MMV007224 and MMV665852, for a prolonged time period to select for resistant parasites. Parasites with resistance to MMV007224 and MMV665852 were found to have an increased copy number of the gene encoding a putative phospholipid flippase, PfATP2. Here we sought to understand how PfATP2 affects parasite sensitivity to the compounds. Using genetically modified parasites, we found that PfATP2 is important for parasite growth and localises to the parasite surface. We showed that increasing the expression level of PfATP2 renders parasites less sensitive to MMV007224 (and the structurally similar molecule MMV665794) and MMV665852, and that decreasing its expression level makes parasites more sensitive to the compounds. We found that the rate by which parasites internalise a fluorescent analogue of phosphatidylserine varied with PfATP2 expression level, consistent with PfATP2 serving as a phospholipid flippase. We tested MMV665794 and MMV007224 in a variety of assays and found that they have multiple effects on the parasite, including inhibition of phosphatidylserine internalisation. Together, the data are consistent with the possibility that inhibition of PfATP2 contributes to parasite killing by the compounds.

## Introduction

*Plasmodium* parasites caused an estimated 263 million cases of malaria and killed 597,000 people in 2023, with *Plasmodium falciparum* causing the majority of deaths [1]. *P. falciparum* parasites are adept at developing resistance to the drugs used against them [2,3], necessitating the continual development of new drugs and investigation of new drug targets. Transporters - proteins or protein complexes (including channels, carriers and pumps) that translocate solutes across membrane bilayers or that move lipids from one membrane leaflet to the other - are overrepresented as drug targets and resistance mediators in *P. falciparum* [4,5]. In a 2018 study, *in vitro* evolution experiments (wherein parasites were exposed to a compound of interest over a prolonged period of time) were performed with 37 antimalarial lead compounds, and the genomes of 262 parasite clones that had acquired resistance were sequenced [4]. Changes (amplification or mutation) in 35 genes were associated with parasite resistance more than once [4]. Among these 35 genes, 17 (i.e., 49%) are known or predicted to encode transporters, despite transporters accounting for only ~ 2.5% of genes in the *P. falciparum* genome [5]. Some of these transporters confer resistance by translocating drugs away from their site of action (e.g., PfMRP1 [6]), while others perform essential functions that are inhibitable by small molecules (i.e., serve as drug targets; e.g., PfATP4 [7,8] and PfFNT [9,10]). It has been suggested that some transporters may double as drug transporters and drug targets (e.g., PfABCI3 [11] and PfCRT [12,13]).

Among the ~ 117 transporters in *P. falciparum*, 13 belong to the P-type ATPase superfamily [5], members of which can be divided into different types [14,15]. P-type ATPases hydrolyse ATP, form a phosphorylated intermediate during their transport cycle, and typically serve as either cation transporters or lipid flippases [15]. One of the *P. falciparum* P-type ATPases, PfATP4 (a Type II P-type ATPase), has emerged as a promising drug target. PfATP4 is believed to extrude Na^+^ ions from the parasite cytosol while importing H^+^ [8]. A host of chemically diverse molecules have been reported to target PfATP4, including the clinical candidate cipargamin [7,8,16–23].

A role for the Type IV P-type ATPase (P4-ATPase) PfATP2 in parasite susceptibility to antimalarial candidates was uncovered for the first time by Cowell *et al.* [4]. P4-ATPases aid in the maintenance of membrane asymmetry by ‘flipping’ specific phospholipids (such as phosphatidylserine (PS) and phosphatidylethanolamine (PE)) from the exoplasmic leaflet of membranes to the cytoplasmic leaflet [reviewed in references 24,25]. Typically, PS and PE are more abundant in the cytoplasmic leaflet of membranes (the inner leaflet in the case of the plasma membrane) as a result of the activity of P4-ATPases, while phosphatidylcholine (PC) is more abundant in the exoplasmic leaflet [26]. A recent study provided evidence that the homologue of PfATP2 in the rodent-infecting malaria parasite *Plasmodium chabaudi* (PcATP2), when expressed alongside the ‘β subunit’ PcCDC50B, functions as a phospholipid flippase [27]. ATP hydrolysis by PcATP2 was stimulated by PS and PE, suggesting that these phospholipids are PcATP2 substrates [27].

Amplification of *pfatp2* (PF3D7_1219600) was associated with *P. falciparum* resistance to the Medicines for Malaria Venture (MMV) compound MMV665852 and was one of several changes observed in parasites resistant to MMV007224 [4]. These two compounds, along with a compound called MMV665794 that is structurally similar to MMV007224, are contained within the MMV’s ‘Malaria Box’ compound collection. In other organisms, changes that reduce the function of P4-ATPases or their β subunits in the plasma membrane have been associated with resistance to phospholipid-like pharmacological agents that are thought to rely on P4-ATPase activity for cellular internalisation [28,29]. In the plant species *Arabidopsis thaliana*, P4-ATPases have been reported to confer resistance to certain (non-phospholipid-like) toxins by mediating their transport via vesicles to vacuoles, where they are sequestered and degraded [30]. The mechanism by which *pfatp2* amplification reduces parasite susceptibility to MMV665852 and MMV007224 is not yet known. In a genome-wide transposon mutagenesis study, PfATP2 was predicted to be essential for the viability of asexual blood stage parasites [31], raising the possibility that PfATP2 is the target of MMV665852 and MMV007224 [4]. The orthologues of PfATP2 in *P. knowlesi* and P. *berghei* have also been predicted to be essential [32,33].

MMV007224, MMV665794 and MMV665852 were found previously by our group to be ‘pH dysregulators’ [10]. The compounds gave rise to a decrease in the pH of the (normally slightly alkaline) parasite cytosol and an increase in the pH of the (normally acidic) digestive vacuole (DV) [10]. The effects of these compounds on parasite pH are consistent with the compounds having protonophore activity (i.e., increasing the permeability of membranes to H^+^). The three MMV compounds (and various synthetic derivatives of them) have also been shown to possess activity against *Schistosoma* worms (e.g., [34–37]).

Here we generated *P. falciparum* lines with altered *pfatp2* expression levels and developed an assay to study PfATP2 activity, in order to determine the function of PfATP2 and understand the mechanism underlying the influence of PfATP2 on parasite susceptibility to MMV665794, MMV007224 and MMV665852.

## Results

### PfATP2 localises to the parasite surface and is important for parasite growth

**Fig 1:**
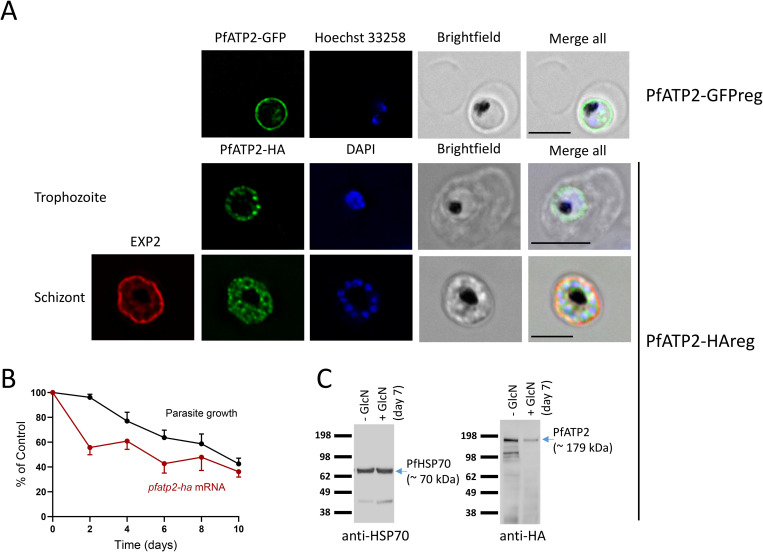
Localisation and knockdown of PfATP2. (**A**) PfATP2-GFP (top) and PfATP2-HA (middle) primarily localise to the parasite surface in intraerythrocytic trophozoite-stage PfATP2-GFPreg and PfATP2-HAreg parasites. In segmenting PfATP2-HAreg parasites (bottom), PfATP2-HA appears to localise to the plasma membrane bounding daughter parasites. Scale bars = 5 µm. Hoechst 33258 and DAPI were used to stain DNA. (**B**) GlcN (5 mM) exposure reduces the level of *pfatp2-ha* mRNA in PfATP2-HAreg parasites (red; with expression compared to the average of two internal controls: 18S rRNA and stRNA) and parasite growth (black; as determined by measuring the parasitemia at the different time points using flow cytometry). The data are from four independent experiments (except for the mRNA data for Days 2, 4 and 8, which are from three independent experiments). In both cases, the data are expressed as a percentage of those obtained for the same parasites that were not treated with GlcN. C. Western blot using antibodies against PfHsp70 (loading control; left) and the HA tag fused to PfATP2 (right) revealed a reduction in PfATP2-HA expression in PfATP2-HAreg parasites treated with 5 mM GlcN for seven days.

To investigate the localisation of PfATP2 and determine whether its expression is important for parasite growth, we made parasite lines (‘PfATP2-GFPreg’ and ‘PfATP2-HAreg’) in which the endogenous *pfatp2* gene was fused at its 3’ end to sequences encoding a GFP tag or a (3×) HA tag and a *glmS* ribozyme (S1 Fig). The *glmS* ribozyme mediates the degradation of the transcript upon parasite exposure to glucosamine (GlcN) [38]. PfATP2-GFP and PfATP2-HA primarily localised to the surface of intraerythrocytic trophozoite-stage parasites, with some internal localisation also evident (**Fig 1A**). We also investigated PfATP2 expression in ring-stage parasites with PfATP2-GFPreg parasites and found that it was expressed (S2 Fig). Subsequent experiments focused on PfATP2-HAreg parasites. To determine whether PfATP2-HA localises to the parasite plasma membrane or the (closely apposed) parasitophorous vacuole membrane, we investigated its localisation in schizont-infected erythrocytes. The parasitophorous vacuole membrane (marked by EXP2 in **Fig 1A**) surrounds the developing daughter parasites, whereas PfATP2-HA appears to be on the plasma membrane surrounding each of the daughter parasites (**Fig 1A**). Upon exposure of PfATP2-HAreg parasites to GlcN (5 mM), we observed a progressive reduction in *pfatp2* mRNA (**Fig 1B**, red) and loss of parasite viability (**Fig 1B**, black). We have shown previously that 5 mM GlcN does not affect the proliferation of wild-type 3D7 parasites under our culture conditions over the same (10 day) time period [39]. Western blotting revealed a reduction in PfATP2-HA protein in PfATP2-HAreg parasites exposed to GlcN (**Fig 1C**). Thus, our data indicate that PfATP2 localises primarily to the parasite plasma membrane and is important for parasite growth.

### PfATP2 expression level affects parasite sensitivity to MMV665794, MMV007224 and MMV665852

**Fig 2:**
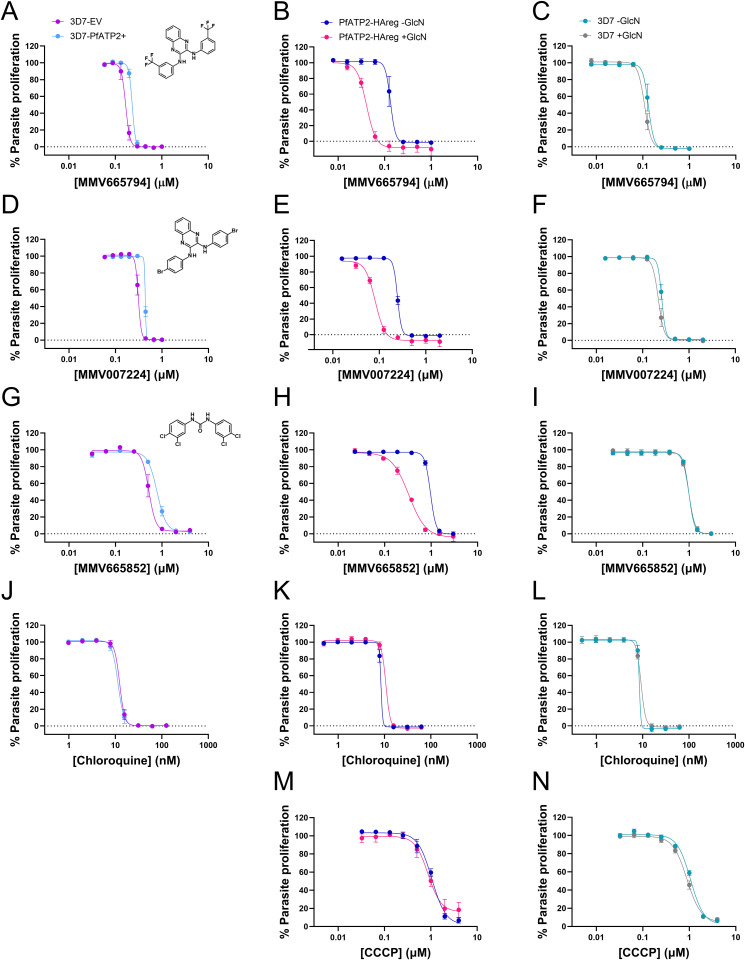
Effect of PfATP2 expression level on parasite response to MMV665794 (A-C), MMV007224 (D-F), MMV665852 (G-I), chloroquine (J-L) and CCCP (M,N). Inhibition of parasite proliferation by the compounds is shown for *pfatp2*-overexpressing parasites (3D7-PfATP2+ ; light blue) and empty vector control parasites (3D7-EV; purple) (panels **A**,**D**,**G**,**J**; CCCP was not tested); PfATP2-HAreg parasites that were not exposed to GlcN (-GlcN; blue) or in which PfATP2-HA was knocked down (PfATP2-HAreg + GlcN; pink) (panels **B**,**E**,**H**,**K**,**M**); and 3D7 parasites that were either exposed (grey) or not exposed (teal) to GlcN (panels **C**,**F**,**I**,**L**,**N**). The data shown are the mean ± SEM from the number of independent experiments indicated in **Table 1**. Where not shown, error bars fall within the symbols. Where present, GlcN (5 mM) was added to cultures four days before the start of the experiment and maintained throughout the 72 h assay.

*pfatp2* amplification has been associated with reduced parasite susceptibility to MMV007224 and MMV665852 [4]. We used genetically modified lines to directly test whether *pfatp2* expression level affects parasite susceptibility to these compounds and MMV665794. First we generated parasites in which an additional copy of the *pfatp2* gene (untagged) is expressed episomally under the control of the *pfcrt* promoter (‘3D7-PfATP2+’). Relative to control parasites transfected with the empty vector (‘3D7-EV’), 3D7-PfATP2+ parasites (at the trophozoite stage) produce 1.5 ± 0.1 fold more *pfatp2* mRNA (mean ± SEM; n = 4). 3D7-PfATP2+ parasites were slightly less susceptible to MMV665794, MMV007224 and MMV665852 (but not to chloroquine) compared to 3D7-EV parasites (**Fig 2**), with the 50% inhibitory concentration (IC_50_) values for the three MMV compounds for 3D7-PfATP2+ parasites being 1.4-1.5 fold higher than those for 3D7-EV parasites (**Table 1**).

**Table 1:** IC_50_ values for growth inhibition of parasites with varying *pfatp2* expression levels by the compounds for which dose-response curves are shown in Fig 2.

	IC_50_ in nM (mean ± SEM) (n)		FC (mean ± SEM)	IC_50_ in nM (mean ± SEM) (n)		FC (mean ± SEM)	IC_50_ in nM (mean ± SEM) (n)		FC (mean ± SEM)
	3D7-EV	3D7-PfATP2+		PfATP2-HAreg -GlcN	PfATP2-HAreg + GlcN		3D7 -GlcN	3D7 +GlcN	
MMV665794	172 ± 13 (5)	233 ± 14 (5)	1.37 ± 0.10***	132 ± 13 (4)	41.4 ± 1.0 (4)	0.323 ± 0.030 ***	132 ± 11 (4)	111 ± 9 (4)	0.84 ± 0.03^ns^
MMV007224	312 ± 14 (3)	428 ± 11 (3)	1.38 ± 0.09**	250 ± 4 (3)	81 ± 1 (3)	0.324 ± 0.004 ***	262 ± 11 (3)	224 ± 17 (3)	0.85 ± 0.05^ns^
MMV665852	532 ± 57 (3)	786 ± 40 (3)	1.50 ± 0.14***	971 ± 42 (3)	341 ± 16 (3)	0.351 ± 0.007 ***	991 ± 41 (3)	993 ± 44 (3)	1.00 ± 0.01^ns^
Chloroquine	13.4 ± 0.8 (9)	12.1 ± 0.9 (9)	0.91 ± 0.04^ns^	9.4 ± 0.5 (5)	11.2 ± 0.8 (5)	1.20 ± 0.07*	10.0 ± 0.9 (5)	9.1 ± 0.4 (5)	0.93 ± 0.07^ns^
CCCP	ND	ND	ND	1040 ± 17 (3)	854 ± 67 (3)	0.82 ± 0.05^ns^	1063 ± 28 (3)	892 ± 42 (3)	0.84 ± 0.03^ns^

The IC_50_ values in nM (mean ± SEM, from the number of independent experiments stated in brackets) are shown. The fold change (FC) values were calculated by dividing the IC_50_ values obtained for 3D7-PfATP2 + , PfATP2-HAreg + GlcN and 3D7 + GlcN parasites by those for 3D7-EV, PfATP2-HAreg -GlcN and 3D7 -GlcN parasites, respectively. A FC > 1 indicates resistance and a FC < 1 indicates hypersensitivity. Two-way ANOVAs with Bonferroni’s multiple comparisons tests were performed using the natural logarithm transformed IC_50_s. ns, *P* ≥ 0.05, *P < 0.05, **P < 0.01, ***P < 0.001. ND, not determined.

We also tested the sensitivity of PfATP2-HAreg parasites in which PfATP2-HA was knocked down to the three MMV compounds, as well as to chloroquine and the well-characterised protonophore CCCP (**Fig 2 and Table 1**). We performed parasite proliferation assays during the window of time in which the majority of GlcN-treated PfATP2-HAreg parasites remained viable despite having a reduced expression of PfATP2-HA (Days 5–7 of GlcN treatment). PfATP2-HA knockdown parasites became hypersensitive to MMV665794, MMV007224 and MMV665852, with the IC_50_ values for the three compounds for PfATP2-HAreg parasites exposed to GlcN being 0.32-0.35 fold those of Control parasites (PfATP2-HAreg parasites not exposed to GlcN). PfATP2-HAreg parasites exposed to GlcN had a slightly higher IC_50_ for chloroquine than those not exposed to GlcN. There was no difference in their sensitivity to CCCP.

To investigate whether the hypersensitivity to the MMV compounds observed with PfATP2-HAreg parasites in the presence of GlcN was a consequence of PfATP2-HA knockdown, and not an off-target effect of GlcN, we also performed parasite proliferation assays with wild-type 3D7 parasites. Exposure of 3D7 parasites to GlcN did not affect their susceptibility to the MMV compounds or to chloroquine or CCCP (**Fig 2 and Table 1**).

We next investigated the speed by which the MMV compounds kill PfATP2-HA knockdown and Control parasites. Consistent with previous findings [40,41] we found that the MMV compounds inhibited parasite growth when applied to intraerythrocytic ring-stage parasites for 24 h (approximately half the time taken for a complete asexual cycle). The compounds gave rise to similar IC_50_ values when they were washed off after 24 h as they did when present throughout the 72 h assays (S3 Fig). The level of hypersensitivity to the compounds observed in parasites in which PfATP2-HA was knocked down was similar regardless of whether or not the compounds were removed after 24 h. After a 24 h exposure, the IC_50_ values for the three compounds for PfATP2-HA knockdown parasites were 0.40-0.43 fold those of Control parasites (S3 Fig).

Having established that *pfatp2* expression level affects parasite response to MMV665794, MMV007224 and MMV665852, we proceeded to investigate the mechanism involved. Our mechanistic studies focused on MMV665794 and MMV007224, due to concerns about the solubility of MMV665852 at the concentrations required for the experiments. MMV665852 and various derivatives of it have been reported to have low aqueous solubility (< 1.6 µg/mL (< 4.6 µM) [42]).

### PfATP2 expression level does not affect pH dysregulation by MMV665794 or MMV007224

MMV665794, MMV007224 and MMV665852 have all been shown to affect the pH in the parasite cytosol (pH_cyt_) and DV (pH_DV_) [10]. pH dysregulation is expected to be detrimental to parasite growth [43], raising the possibility that the three MMV compounds kill parasites via this mechanism. Given that *pfatp2* expression level alters the antiplasmodial potency of the compounds, we reasoned that if pH dysregulation were the primary mechanism of action of the compounds, this process should be impacted by the *pfatp2* expression level. We therefore investigated whether PfATP2 expression level affects parasite pH regulation or the potency by which the MMV compounds dysregulate pH_cyt_.

**Fig 3:**
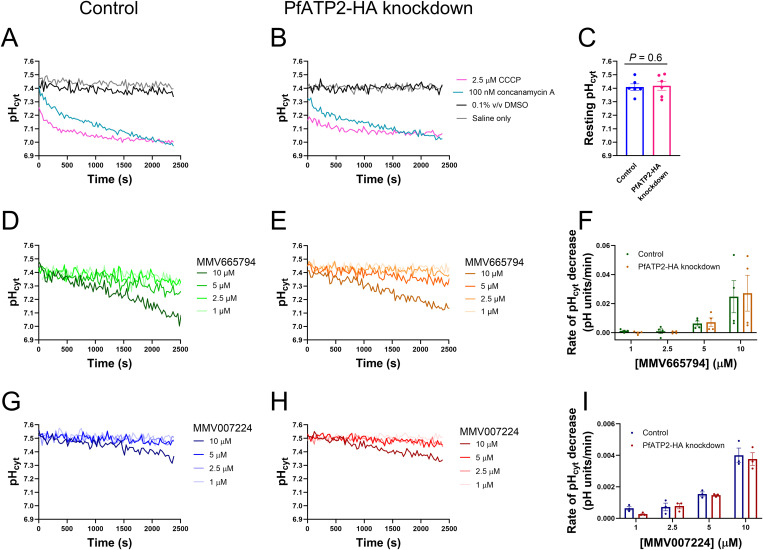
A reduced expression level of PfATP2-HA does not affect the resting pH_cyt_ in *P. falciparum* parasites or pH_cyt_ dysregulation by MMV665794 or MMV007224. Isolated trophozoite-stage PfATP2-HAreg parasites (Control parasites (-GlcN) or PfATP2-HA knockdown parasites (from cultures exposed to 5 mM GlcN for four days)) suspended in pH 7.1 Physiological Saline at 37˚C were exposed to either the protonophore CCCP, the V-type H^+^ ATPase inhibitor concanamycin A, MMV665794 or MMV007224 (at the concentrations indicated), solvent alone (black; 0.1% v/v DMSO) or Physiological Saline only (grey). GlcN was not present during the measurements. (**A**,**B**,**D**,**E**,**G**,**H**) Traces from single experiments with Control parasites (**A**,**D**,**G**) or PfATP2-HA knockdown parasites (**B**,**E**,**H**), representative of at least three independent experiments. (**C**) The resting pH_cyt_ of Control parasites (blue) and PfATP2-HA knockdown parasites (pink) averaged from the first 2000 s of solvent control (0.1% v/v DMSO) traces. The data are from six independent experiments. The resting pH_cyt_ of Control and PfATP2-HA knockdown parasites were compared using a paired t-test, yielding a *P* value of 0.6. (**F,I)** The rate of pH_cyt_ decrease was estimated from the initial linear portions of the traces for each concentration of MMV665794 (**F**; first 300-2400 s; n = 4) and MMV007224 (**I**; first 2400 s; n = 3). (**C**,**F**,**I**) Control parasites and PfATP2-HA knockdown parasites were tested concurrently in each experiment. The symbols show the data from individual experiments; the bars and error bars show the mean ± SEM. Note that the y-axis ranges are different in panels **F** and **I**. Two-way ANOVAs performed with the data shown in Panels **F** and **I** revealed a significant effect of the concentration of the MMV compound on the rate of pH_cyt_ decrease (*P* ≤ 0.04) but no significant effect of PfATP2-HA knockdown (*P* ≥ 0.5).

The first series of experiments was performed with isolated trophozoite-stage PfATP2-HAreg parasites that were loaded with the pH-sensitive fluorescent dye BCECF and suspended at 37˚C in pH 7.1 Physiological Saline. Control parasites (-GlcN) and PfATP2-HA knockdown parasites (from cultures exposed to 5 mM GlcN for four days in the lead up to the experiment) both maintained a similar, slightly alkaline pH_cyt_ when suspended in pH 7.1 Physiological Saline (**Fig 3A–C**). For Control parasites the pH_cyt_ was 7.41 ± 0.02 and for PfATP2-HA knockdown parasites the pH_cyt_ was 7.42 ± 0.03 (mean ± SEM, n = 6; *P* = 0.6, paired t-test). Upon inhibition of the V-type H^+^ ATPase, which serves as the parasite’s primary regulator of pH_cyt_ [44], with concanamycin A (100 nM) there was a decrease in pH_cyt_ in Control and PfATP2-HA knockdown parasites, as expected from previous studies with different *P. falciparum* strains (e.g., [22,43]). The protonophore CCCP (2.5 µM) also gave rise to a rapid decrease in pH_cyt_ in Control and PfATP2-HA knockdown parasites (**Fig 3A and 3B**), consistent with previous studies performed with other *P. falciparum* strains [45].

We next investigated the effects of MMV665794 and MMV007224 on pH_cyt_ in Control and PfATP2-HA knockdown parasites. In both parasite types, a decrease in pH_cyt_ was readily detectable at concentrations ≥ 5 µM for MMV665794 (**Fig 3D and 3E**) and 10 µM for MMV007224 (**Fig 3G and 3H**). We estimated the rate of pH_cyt_ decrease from the initial linear portions of the traces. While there was some variability between experiments, the rate of pH_cyt_ decrease was observed to increase with increasing concentrations of the compounds (**Fig 3F and 3I**). There was no significant effect of PfATP2-HA knockdown on the rate by which the pH_cyt_ decreased in the presence of the compounds.

### MMV665794 and MMV007224 act like protonophores

Protonophores and V-type H^+^ ATPase inhibitors both give rise to a decrease in pH_cyt_ and an increase in pH_DV_ (the same effects observed for MMV665794, MMV007224 and MMV665852 [10]). The V-type H^+^ ATPase localises to the parasite plasma membrane, transporting H^+^ out of the parasite, and to the DV membrane, pumping H^+^ into this organelle [44,46,47]. The next series of experiments was designed to test whether MMV665794 and MMV007224 have protonophore activity (i.e., render membranes more permeable to H^+^), and if so, to determine whether PfATP2 expression level (in the lead up to the experiment) affected the properties of the plasma membrane in such a way as to influence their potencies as protonophores.

In these experiments, parasites were depleted of ATP (by incubation in Glucose-free Saline) and suspended at time zero in (glucose-free) Low Cl^-^ Saline. The parasite has an acid-loading Cl^-^ transport process, which, under physiological conditions, imports Cl^-^ ions and H^+^ equivalents [48]. However, when parasites are suspended in a low Cl^-^ saline (creating an outward Cl^-^ gradient across the parasite plasma membrane), the direction of transport is reversed such that Cl^-^ and H^+^ equivalents exit the parasite. Under these conditions, protonophores such as CCCP give rise to an increase in the rate of cytosolic alkalinisation, whereas V-type H^+^ ATPase inhibitors do not [49]. ATP-hydrolysing transporters including the V-type H^+^ ATPase and PfATP2 are not expected to be active in the ATP-depleted parasites used in these assays.

**Fig 4:**
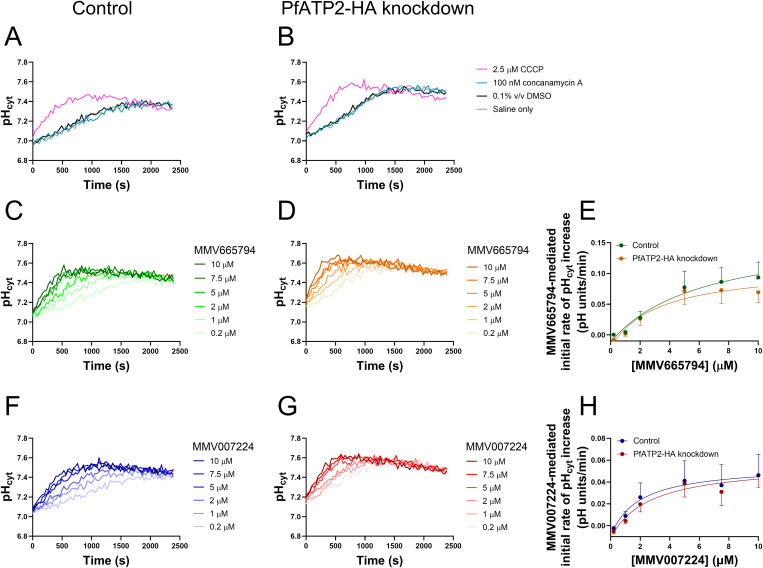
MMV665794, MMV007224 and the protonophore CCCP, but not the V-type H^+^ ATPase inhibitor concanamycin A, increased the rate of cytosolic alkalinisation observed upon creation of an outward Cl^-^ gradient in Control parasites and PfATP2-HA knockdown parasites. (**A**-**D**,**F**,**G**) Representative traces from single experiments with Control parasites (PfATP2-HAreg -GlcN; **A**,**C**,**F**) and PfATP2-HA knockdown parasites (PfATP2-HAreg exposed to 5 mM GlcN for four days; **B**,**D**,**G**), suspended at time 0 in Low Cl^-^ Saline (37˚C). GlcN was not present during the measurements. **(A,B)** Parasites were exposed to either the protonophore CCCP (pink; 2.5 µM), the V-type H^+^ ATPase inhibitor concanamycin A (teal; 100 nM), solvent alone (black; 0.1% v/v DMSO) or Low Cl^-^ Saline only (grey). Each of the four conditions were included in at least two independent experiments, yielding similar results. (**C**,**D**,**F**,**G**) Parasites were exposed to MMV665794 (**C**,**D**) or MMV007224 **(F,G)**, with each compound tested at concentrations of 0.2, 1, 2, 5, 7.5 and 10 µM. The concentration of solvent (DMSO) was 0.1% v/v in each case. (**E,H)** The rate of pH_cyt_ increase was estimated from the initial linear portions of the traces for each concentration of MMV665794 (**E**; first 150-550 s) and MMV007224 (**H**; first 200-1000 s). Within each experiment, the rate of pH_cyt_ increase calculated for the 0.1% v/v DMSO control trace (first 800-1100 s) was subtracted from the rate of pH_cyt_ increase calculated for each concentration of each compound. Control parasites and PfATP2-HA knockdown parasites were tested concurrently in each experiment. The data are the mean ± SEM from five independent experiments. Note that the y-axis ranges are different in panels **E** and **H**. Two-way ANOVAs performed with the data shown in panels **E** and **H** revealed a significant effect of the concentration of the MMV compound on the initial rate of pH_cyt_ increase (*P* ≤ 0.0004) but no effect of PfATP2-HA knockdown (*P* ≥ 0.3).

In both Control parasites (**Fig 4A**) and PfATP2-HA knockdown parasites (**Fig 4B**), the protonophore CCCP (2.5 µM) increased the rate of cytosolic alkalinisation following suspension of the parasites in Low Cl^-^ Saline. The V-type H^+^ ATPase inhibitor concanamycin A (100 nM) did not affect the rate of cytosolic alkalinisation (**Fig 4A and 4B**) – its traces overlapped with those for the solvent control (0.1% v/v DMSO) and Saline only control. This is consistent with the V-type H^+^ ATPase being inactive under the conditions of the experiment.

We next investigated the effects of MMV665794 and MMV007224 on the rate of cytosolic alkalinisation in Control and PfATP2-HA knockdown parasites. Both compounds caused a dose-dependent increase in the rate of cytosolic alkalinisation in Control parasites (**Fig 4C and 4F**) and PfATP2-HA knockdown parasites (**Fig 4D and 4G**) suspended in Low Cl^-^ Saline, consistent with the compounds having protonophore activity. For both compounds, the rate of cytosolic alkalinisation increased as the concentration of the compound was increased between 0.2 µM – 5 µM, with the rate of alkalinisation then remaining similar when the concentration was increased further. The initial rate of pH_cyt_ increase mediated by the compounds was higher for MMV665794 (~ 0.07-0.09 pH units/min at concentrations ≥ 5 µM) than for MMV007224 (~ 0.04-0.05 pH units/min at concentrations ≥ 5 µM) (**Fig 4E and 4H**). There was no effect of PfATP2-HA knockdown on the initial rate of pH_cyt_ increase measured in the presence of the compounds.

In summary, we confirmed that MMV665794 and MMV007224 decrease pH_cyt_ (**Fig 3**) and provided evidence that they do so by acting as protonophores (**Fig 4**). Reducing the level of PfATP2-HA expression did not affect the parasite’s resting pH_cyt_ or the protonophore activities of the compounds, suggesting that pH dysregulation may not be the primary mode by which the compounds kill parasites.

### PfATP2 expression level affects NBD-PS internalisation

To determine whether MMV665794 and MMV007224 kill parasites by targeting PfATP2, we first needed to develop an assay to study PfATP2 activity. To investigate whether PfATP2 serves as a phospholipid flippase on the parasite plasma membrane, we measured the internalisation of a fluorescent PS analogue (NBD-PS) into saponin-isolated trophozoite-stage parasites with varying PfATP2 expression levels. We focused on NBD-PS as it is often a substrate of P4-ATPases and a previous study reported that NBD-PS internalisation by isolated *P. falciparum* parasites was sensitive to the P-type ATPase inhibitor vanadate (implicating a P-type ATPase in the uptake process) [50]. Parasites were incubated with NBD-PS for varying lengths of time, with BSA then used to scavenge the NBD-PS that remained outside the cells or in the extracellular leaflet of the parasite plasma membrane (allowing us to measure the NBD-PS that had been internalised). We also attempted to measure the uptake of NBD-PE by isolated parasites. However, the BSA extraction strategy we used successfully to remove NBD-PS from the outer leaflet of the plasma membrane did not appear to be effective for NBD-PE (S4 Fig).

**Fig 5:**
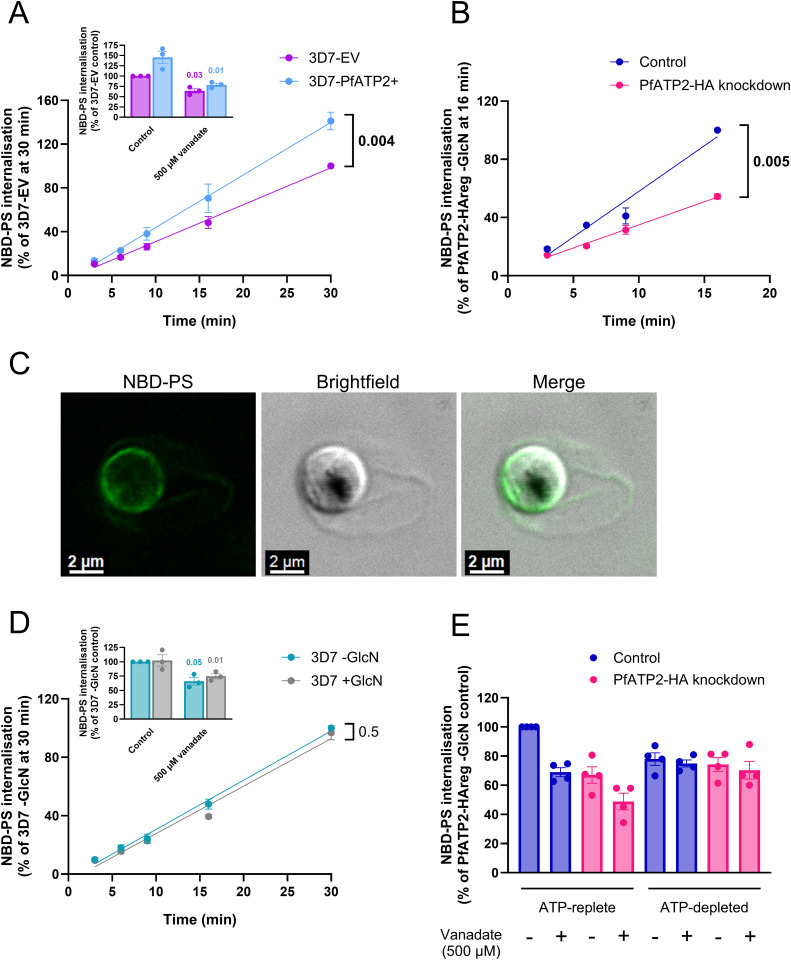
NBD-PS internalisation by parasites is affected by the expression level of PfATP2, the P-type ATPase inhibitor vanadate and the availability of ATP. (**A**,**B**,**D**) NBD-PS internalisation over time in 3D7-PfATP2+ (light blue) and 3D7-EV (purple) parasites **(A)**, PfATP2-HA knockdown (pink; from cultures exposed to 5 mM GlcN for two days) or Control (blue; -GlcN) parasites **(B)**; wild-type 3D7 parasites from cultures that were exposed to 5 mM GlcN for two days (+GlcN; grey) or not exposed to GlcN (-GlcN; teal) **(D)**. The data are the mean (± SEM) from five **(A)**, three (**B**) or four (**D**) independent experiments. **C**. Confocal images of an isolated PfATP2-HAreg parasite (-GlcN) after a 9 min incubation with NBD-PS at 15˚C. In **A**-**D**, NBD-PS internalisation was measured in isolated trophozoite-stage parasites suspended in pH 7.1 Physiological Saline at 15˚C. In **A**, **B** and **D**, the results of ratio paired t-tests, performed with the slopes obtained when lines were fit to the pre-normalised data (NBD-PS fluorescence (geometric mean)), are shown, with *P* values indicating a significant difference (*P* < 0.05) shown in bold. **E**. NBD-PS uptake over 9 min at 15˚C in isolated PfATP2-HA knockdown or Control parasites, in the absence (solvent control) or presence of vanadate (500 µM). Parasites were either ATP-replete (and suspended in pH 7.1 Physiological Saline) or ATP-depleted (and suspended in Glucose-free Saline). A three-way ANOVA was performed on the natural logarithm transformed pre-normalised data. The effects of PfATP2-HA knockdown and vanadate were both dependent on the presence of ATP (*P* = 0.02 and *P* = 0.04, respectively). The pairwise comparisons for which *P* values are provided in the text are from a post hoc Tukey test. **Insets**: NBD-PS uptake (9 min, 15°C) in 3D7-PfATP2+ and 3D7-EV parasites (**A inset**) and 3D7 parasites (+GlcN or -GlcN; **D inset**) in the absence (solvent control) or presence of vanadate (500 µM). The P values are from ratio paired t-tests performed with the pre-normalised data. **E** and **insets**: The symbols show the data from individual experiments; the bars and error bars show the mean ± SEM. The data are from three (**insets**) or four (**E**) independent experiments, in which all the conditions/parasite types shown were tested concurrently. In all panels except **C**, the data are expressed as a percentage of the NBD-PS fluorescence (geometric mean) measured in the parasites/condition indicated on the y axis. GlcN was not present when parasites were exposed to NBD-PS.

*pfatp2* overexpressing 3D7-PfATP2+ parasites internalised more NBD-PS than 3D7-EV control parasites over the course of 30 min (**Figs 5A and** S5A), consistent with PfATP2 contributing to PS internalisation by the parasites. We did not detect any difference in the growth rate of 3D7-PfATP2+ and 3D7-EV parasites (S6A Fig), and our synchronisation strategy ensured that isolated trophozoites from the two cell populations had very similar mean volumes (S6B Fig).

PfATP2-HA knockdown parasites (from cultures exposed to 5 mM GlcN for two days in the lead-up to the experiment) internalised less NBD-PS over time than Control parasites (-GlcN) (**Figs 5B and** S5B). Visualisation of Control parasites after a 9 min exposure to NBD-PS showed that the majority of the fluorescence was at the parasite surface, consistent with parasite plasma membrane processes playing a major role in NBD-PS internalisation (**Fig 5C**). To confirm that it was PfATP2-HA knockdown that led to the reduction in NBD-PS internalisation rather than an off-target effect of GlcN exposure, we also performed measurements with wild-type 3D7 parasites. Exposure of cultures to 5 mM GlcN for two days in the lead-up to the experiment had no effect on NBD-PS internalisation by 3D7 parasites (**Figs 5D and** S5C).

Unless stated otherwise, we used a two day (~ 48 h) exposure to 5 mM GlcN in the lead up to our NBD-PS uptake experiments (shorter than the exposure times used in the experiments described in the previous sections) to minimise the difference in parasite stage between the PfATP2-HA knockdown and Control parasites. However, using a Coulter Counter, we found that (saponin-isolated) PfATP2-HAreg parasites from cultures that had been exposed to GlcN for two days had a mean cell volume that was reduced to 79 ± 6% (mean ± SEM, n = 5) of that measured for PfATP2-HAreg parasites not exposed to GlcN (S7A Fig). To investigate whether the smaller cell volume was a result of PfATP2-HA knockdown or a different effect of GlcN, we also measured the volume of 3D7 parasites from cultures that had been exposed to GlcN for two days. We found that GlcN had an effect on the mean volume of 3D7 parasites (although this was less pronounced than that observed for PfATP2-HAreg parasites), reducing it to 89 ± 2% (mean ± SEM, n = 9) of that measured in 3D7 parasites that had not been exposed to GlcN (*P* = 0.002, ratio paired t-test). The basis for the effect of GlcN on parasite volume is not clear; in 3D7 parasites it is not associated with a reduction in parasitemia [39] or a decrease in NBD-PS internalisation (**Fig 5D**).

To further investigate whether the smaller volume of PfATP2-HA knockdown parasites might account for their reduced uptake of NBD-PS relative to Control parasites, we also performed experiments in which PfATP2-HAreg parasites were only exposed to GlcN for one day (~ 24 h). The mean volume of isolated PfATP2-HAreg parasites from cultures that had been exposed to GlcN for one day was 92 ± 1% (mean ± SEM, n = 3) of that observed for PfATP2-HAreg parasites not exposed to GlcN (S7A Fig). However, the reduction in NBD-PS uptake observed in PfATP2-HAreg parasites that were exposed to GlcN for either one or two days was very similar, with parasites internalising 28–30% less NBD-PS (at the 9 min time point) than Control parasites in both cases (S7B Fig). Thus, the reduction in NBD-PS uptake by PfATP2-HA knockdown parasites cannot be explained by a decrease in cell volume.

As an additional control, we tested PfABCI3-HAreg parasites (made previously [39]) that had been treated with GlcN for one or two days. The effect of GlcN on the volume of PfABCI3-HAreg parasites was similar to that observed for PfATP2-HAreg parasites (S7C Fig). Unlike PfATP2-HAreg parasites exposed to GlcN for one day, PfABCI3-HAreg parasites exposed to GlcN for one day did not internalise less NBD-PS than PfABCI3-HAreg parasites that had not been exposed to GlcN (S7D Fig). However, NBD-PS internalisation by PfABCI3-HAreg parasites that had been exposed to GlcN for two days was 21 ± 2% lower (mean ± SEM, n = 3) than that observed for PfABCI3-HAreg parasites not exposed to GlcN (S7D Fig). Of note, PfABCI3-HAreg parasites exposed to GlcN lose viability more quickly than PfATP2-HAreg parasites exposed to GlcN (with parasite growth after a two day exposure to GlcN reduced to 87 ± 1% of control levels in the former [39] and 96 ± 2% of control levels in the latter (mean ± SEM, n = 4; **Fig 1B**)). Whether the finding of reduced NBD-PS uptake by PfABCI3-HAreg parasites after a two day (but not a one day) exposure to GlcN is a result of reduced parasite viability or a direct or indirect effect of PfABCI3 on phospholipid transport is not clear.

Measurements of phospholipid uptake are typically performed at temperatures below 20˚C in order to reduce uptake by endocytosis [51]. Unless stated otherwise, our NBD-PS uptake assays were performed at 15˚C. However, we also investigated NBD-PS uptake at the temperature normally experienced by the parasite (37˚C). The overall uptake of NBD-PS was higher at 37˚C than at 15˚C, but the effect of knocking down PfATP2-HA on the proportion of NBD-PS taken up was similar, with internalisation reduced by 32 ± 6% at the 9 min time point at 37˚C in the PfATP2-HA knockdown parasites compared to the Control parasites (mean ± SEM, n = 7; *P* = 0.004, ratio paired t-test using pre-normalised data; S8A and S8B Fig).

To gain further insight into the mechanisms responsible for parasite NBD-PS uptake, we investigated NBD-PS internalisation (at 15˚C at the 9 min time point) by PfATP2-HA knockdown and Control parasites in the presence and absence of the P-type ATPase inhibitor vanadate (500 µM), and under conditions in which the parasites were ATP-replete or depleted of ATP (by incubation in Glucose-free Saline). In ATP-replete parasites, the knockdown of PfATP2-HA or presence of vanadate both resulted in a significant reduction in NBD-PS internalisation (to 67–69% of Control levels; **Figs 5E and** S5D). In ATP-depleted parasites, neither the knockdown of PfATP2-HA nor the presence of vanadate affected NBD-PS internalisation, as expected (**Fig 5E**).

Vanadate also reduced NBD-PS internalisation in ATP-replete PfATP2-HA knockdown parasites (*P* = 0.006; **Fig 5E**). This could be a result of the inhibition of PfATP2-HA, as residual expression of the protein is expected in the knockdown parasites (**Fig 1B and 1C**), and/or other ATP-dependent internalisation mechanism(s). NBD-PS internalisation was lower in PfATP2-HA knockdown parasites treated with vanadate than in Control parasites treated with vanadate (*P* = 0.04; **Fig 5E**). We also found that vanadate showed greater inhibition of NBD-PS uptake at 37˚C than at 15˚C in Control parasites (but not PfATP2-HA knockdown parasites) (S8B Fig). This raises the possibility that vanadate may not be fully soluble at 15˚C, and that at this temperature the concentration in solution was not sufficient to fully inhibit P-type ATPase activity in parasites with a normal expression level of PfATP2-HA. The finding that NBD-PS internalisation in ATP-depleted parasites was somewhat higher than in ATP-replete parasites in which PfATP2-HA activity was expected to be minimal (e.g., PfATP2-HA knockdown parasites exposed to vanadate (*P* ≤ 0.02; **Fig 5E**)) raises the possibility that the activity of ATP-independent internalisation mechanism(s) is greater in ATP-depleted parasites than in ATP-replete parasites.

We also tested vanadate for its effect on NBD-PS uptake by (ATP-replete) 3D7-PfATP2+ and 3D7-EV parasites (**Fig 5A inset**), as well as 3D7 parasites from cultures that were either exposed to GlcN in the lead-up to the experiment or not exposed to GlcN (**Fig 5D inset**). Vanadate reduced NBD-PS internalisation (by 27–45%) in each case.

A previous study identified PfCDC50C as the interacting partner of PfATP2 but reported no change in NBD-PS uptake when PfCDC50C was knocked out [52]. Patel *et al.* [52] performed their experiments with parasitised erythrocytes, which were incubated with NBD-PS for 30 min at 37˚C. We investigated whether an effect of PfATP2-HA knockdown on NBD-PS uptake could be detected in infected erythrocytes under these conditions. We found that a two day exposure to GlcN led to a significant decrease in overall NBD-PS internalisation by erythrocytes infected with mature trophozoite-stage PfATP2-HAreg parasites, albeit smaller than that seen for isolated parasites, with NBD-PS uptake in parasitised erythrocytes that had been exposed to GlcN for two days being 78 ± 4% of that observed for the -GlcN control cells (mean ± SEM, n = 4; S9A Fig). In line with our results with isolated parasites, and with previous data obtained with 3D7 parasites and parasitised erythrocytes [50], vanadate significantly inhibited NBD-PS uptake by erythrocytes infected with Control and PfATP2-HA knockdown parasites (S9A Fig).

We also analysed NBD-PS uptake by uninfected erythrocytes (co-cultured with parasitised erythrocytes, which were distinguishable from the parasitised erythrocytes with flow cytometry). Prior exposure to GlcN had no effect on the NBD-PS fluorescence measured in uninfected erythrocytes at the 30 min timepoint (S9B Fig). Vanadate appeared to cause some inhibition of NBD-PS uptake by uninfected erythrocytes (as reported previously [50]), although in our study this was not statistically significant (S9B Fig).

### MMV665794 and MMV007224 inhibit NBD-PS uptake by parasites

**Fig 6:**
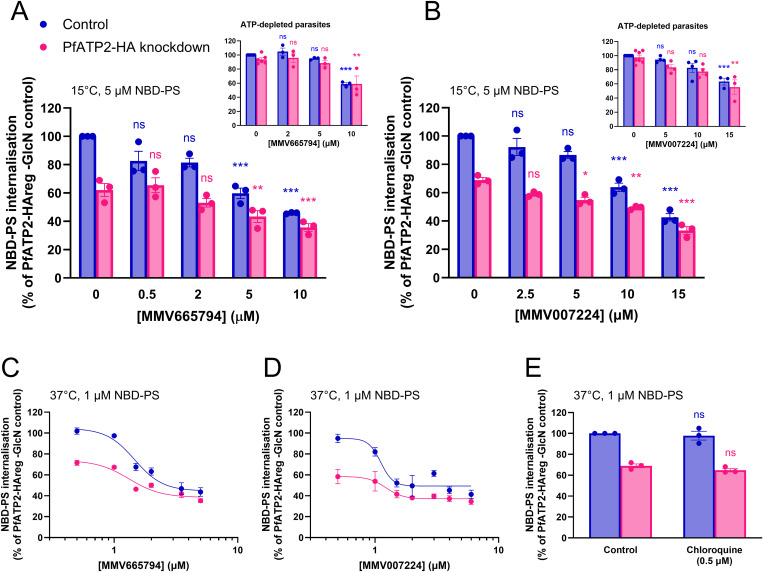
NBD-PS internalisation by parasites is inhibited by MMV665794 and MMV007224 but not by chloroquine. NBD-PS internalisation was measured in isolated trophozoite-stage PfATP2-HA knockdown (pink; from cultures exposed to 5 mM GlcN for two days) or Control (blue; PfATP2-HAreg -GlcN) parasites. The parasites were either ATP-replete (and suspended in pH 7.1 Physiological Saline; all main panels) or ATP-depleted (and suspended in Glucose-free Saline; **insets**). The complete experiments from which the data in the **insets** are derived (including data for the ATP-replete parasites tested in parallel) are shown in S11 Fig. The experiments were performed either at 15°C with 5 µM NBD-PS over 9 min (**A**,**B**) or at 37°C with 1 µM NBD-PS over 30 min **(C-E)**, in the absence (solvent control) or presence of the compounds and concentrations indicated. The data are expressed as a percentage of the NBD-PS fluorescence (geometric mean) measured in the parasites/condition indicated on the y axis. (**A**,**B**,**E**) The data are from at least three independent experiments. With the exception of the **insets**, all the conditions/parasite types shown within one panel were tested concurrently. The symbols show the data from individual experiments; the bars and error bars show the mean ± SEM. In **A** and **B**, statistical comparisons were made for each parasite type between the pre-normalised (NBD-PS fluorescence (geometric mean)) data for different concentrations of the MMV compounds and the data for the solvent control using lognormal one-way ANOVAs with post hoc Dunnett’s tests. In **E**, ratio paired t-tests using the pre-normalised data were performed (ns, *P* > 0.5; *P < 0.5; **P < 0.01; ***P < 0.001). **(C,D)** The data are the mean ± SEM from four independent experiments (**C**; except for the 1.5 µM (n = 2) and 1 µM (n = 3) concentrations) or three independent experiments (**D**; except for the 3, 1.5 and 0.5 µM concentrations, which are n = 2).

To investigate whether MMV665794 and MMV007224 target PfATP2, we tested a range of concentrations of the compounds (after 9 min at 15˚C) for their effects on NBD-PS internalisation. In PfATP2-HAreg Control parasites (-GlcN), MMV665794 significantly inhibited NBD-PS internalisation at concentrations ≥ 5 µM (**Figs 6A and** S5E), and MMV007224 did so at concentrations ≥ 10 µM (**Figs 6B and** S5F). These compounds also inhibited NBD-PS internalisation in PfATP2-HA knockdown parasites (**Fig 6A and 6B**). This could be a result of the MMV compounds inhibiting residual PfATP2-HA and/or an internalisation mechanism other than PfATP2 (investigated further below).

We tested the protonophore CCCP to investigate whether dysregulation of parasite pH_cyt_ might indirectly affect NBD-PS internalisation. CCCP (tested at 100 nM and 5 µM at 15˚C) did not show evidence for the inhibition of NBD-PS internalisation in PfATP2-HA knockdown or Control parasites (S10 Fig). Thus, the effects of the MMV compounds on NBD-PS internalisation are unlikely to stem from their effects on pH_cyt_. When tested at the higher concentration, CCCP appeared to increase fluorescence. Whether this resulted from an increase in NBD-PS uptake or a contribution to the fluorescence from CCCP itself is not clear.

To gain further insight into whether MMV665794 and MMV007224 reduce NBD-PS internalisation via inhibition of PfATP2, we compared the effects of the compounds on NBD-PS internalisation in ATP-replete and ATP-depleted parasites. If the compounds inhibit NBD-PS uptake solely via inhibition of PfATP2, they should have no effect in ATP-depleted parasites. When tested at 2 µM and 5 µM (MMV665794; S11B Fig) or 5 and 10 µM (MMV007224; S11C Fig), the compounds did not significantly inhibit NBD-PS internalisation by ATP-depleted parasites (**Fig 6A and 6B** **insets**), consistent with the compounds displaying some selectivity for inhibition of ATP-dependent NBD-PS internalisation. However, when tested at 10 µM (MMV665794) or 15 µM (MMV007224), both compounds appeared to reduce NBD-PS internalisation by both ATP-replete and ATP-depleted parasites (**Figs 6A**, **6B** **insets** and S11A). The data raise the possibility that high concentrations of MMV665794 and MMV007224 inhibit an ATP-independent NBD-PS uptake mechanism, although we cannot rule out the possibility that they cause a reduction in fluorescence by other means (e.g., via an effect on fluorescence or an effect on membranes that alters NBD-PS incorporation or the action of BSA).

The concentrations of MMV665794 and MMV007224 required to significantly inhibit ATP-dependent NBD-PS internalisation in our initial assays were ~ 40-fold higher than the IC_50_ values of the compounds against PfATP2-HA Control parasites in 72 h growth assays. We investigated the possibility that NBD-PS and the MMV compounds compete for binding to PfATP2, and that the potency of the compounds is affected by the NBD-PS concentration used in the assays. To do this, we performed another series of assays in which the NBD-PS concentration in the reaction mixtures was reduced (from 5 µM) to 1 µM. To increase the resolution of our assays we tested additional low concentrations of the compounds (with all concentrations tested being lower than those observed to inhibit NBD-PS uptake by ATP-depleted parasites) and performed the assays under conditions in which NBD-PS uptake is maximised (30 min at 37˚C; S5G–I Fig).

In these assays, the difference in NBD-PS internalisation by PfATP2-HA knockdown and Control parasites was pronounced in the absence of the MMV compounds and at the lowest concentrations tested, then diminished as the concentrations of the compounds increased (**Fig 6C and 6D**). In both PfATP2-HA knockdown and Control parasites, the higher concentrations of the compounds (3.5-5 µM for MMV665794 and 4–6 µM for MMV007224) inhibited NBD-PS uptake to 34–47% of that observed in Control parasites in the absence of compound. The remaining NBD-PS internalisation may be contributed by pathways other than PfATP2 (including ATP-independent mechanisms). From dose-response curves fitted to the data shown in **Fig 6C and 6D**, we estimated half-maximal effective concentration (EC_50_) values for MMV665794 of 1.3 µM (PfATP2-HA knockdown parasites) and 1.5 µM (Control parasites). For MMV007224 the values were 1.2 µM (PfATP2-HA knockdown parasites) and 1.1 µM (Control parasites). These values are 5–11 fold higher than the IC_50_ values of the compounds against PfATP2-HA Control parasites in 72 h growth assays; it is possible that further reducing the NBD-PS concentration would result in lower EC_50_ values for the compounds in the NBD-PS uptake assays.

As a control, we also tested the antimalarial chloroquine for its effect on NBD-PS internalisation under the same conditions and at a concentration ~ 50-fold higher than its IC_50_ against PfATP2-HA Control parasites in 72 h growth assays (0.5 µM). Chloroquine did not affect NBD-PS uptake by PfATP2-HA Control or knockdown parasites (**Fig 6E**).

While the experiments for which data are shown in **Fig 6A and 6B** were performed at 15˚C, similar effects for MMV665794 (10 µM) and MMV007224 (15 µM) were observed at 37˚C (S12A Fig). Our detailed studies on the effects of the MMV compounds focused on PfATP2-HAreg parasites; only single high concentrations (10 µM MMV665794 and 15 µM MMV007224) were tested against 3D7-PfATP2+ and 3D7-EV parasites (S12B Fig), and wild-type 3D7 parasites (+ and - GlcN) (S12C Fig), with a reduction in NBD-PS fluorescence observed in all cases.

We also investigated the effects of MMV665794 and MMV007224 on NBD-PS uptake by parasitised erythrocytes and (co-cultured) uninfected erythrocytes. When tested at 10 µM (MMV665794) or 15 µM (MMV007224), the compounds significantly decreased the NBD-PS fluorescence measured in both parasitised erythrocytes (S13A Fig) and uninfected erythrocytes (S13B Fig). We then investigated whether the MMV compounds affect NBD-PS uptake by infected erythrocytes and uninfected erythrocytes at a lower concentration (2 µM). These experiments were performed with cultures of young trophozoites and mature trophozoites, to investigate whether the maturity of the parasites had an impact on the effects of GlcN or the MMV compounds on NBD-PS uptake. Exposure of cultures to GlcN for two days in the lead-up to the experiment had no effect on NBD-PS uptake by uninfected erythrocytes, but led to a small but significant reduction in NBD-PS uptake by parasitised erythrocytes, regardless of whether the trophozoites were mature or younger (S13C–F Fig). When tested at 2 µM, both MMV compounds gave rise to a small (8–17%) decrease in the NBD-PS fluorescence measured at the 30 min timepoint in erythrocytes infected with young and mature trophozoites, as well as uninfected erythrocytes (S13C–F Fig).

Together, the data are consistent with the possibility that MMV665794 and MMV007224 inhibit PfATP2. The concentrations required to inhibit ATP-dependent NBD-PS internalisation are lower than those needed to observe any effect on ATP-depleted parasites. In experiments performed with 1 µM NBD-PS at 37°C, low micromolar concentrations of both MMV compounds inhibited NBD-PS internalisation by ATP-replete parasites, with the level of uptake observed in PfATP2-HA knockdown and Control parasites converging as the concentrations of the MMV compounds increased. A modest effect of 2 µM MMV665794 and MMV007224 on NBD-PS uptake was also observed in parasitised and uninfected erythrocytes. At concentrations ≥ 10 µM, the compounds appear to inhibit NBD-PS uptake process(es) that are active in ATP-depleted parasites or otherwise affect the assays in such a way as to reduce NBD-PS fluorescence.

## Discussion

This study provides evidence that PfATP2 serves as an important phospholipid flippase on the *P. falciparum* plasma membrane, with PS being a substrate of the transporter. However, measurements of the activity of the protein in purified form, or expressed (with its β subunit) in a heterologous expression system, would be required for direct confirmation. PfATP2 has the signature motifs characteristic of a P4-ATPase [53] and contains a Q residue in transmembrane helix 1 equivalent to Q88 in human ATP8A1, which interacts with the headgroup of PS and is critical for PS transport [26,54–57].

Most P4-ATPases interact with a non-catalytic β subunit [26]. In *P. falciparum* there are three candidate ‘cell division control 50’ (CDC50) proteins. One of these, PfCDC50C, was recently shown to interact with PfATP2 in parasites and to be essential for the viability of blood-stage parasites [52]. PfCDC50A was not expressed in asexual parasites and PfCDC50B interacted instead with guanylyl cyclase α (GCα) [52]. Studies with the related apicomplexan parasite *Toxoplasma gondii* yielded consistent findings. *T. gondii* expresses two ATP2 proteins (ATP2A and ATP2B) [53,58], both of which localise at least in part to the plasma membrane, with enrichment observed at the apical end in the case of ATP2B [53]. Both proteins have been found to interact with the homologue of PfCDC50C (CDC50.4) [58,59]. In a study with recombinantly expressed *P. chabaudi* proteins, PcATP2 was found to form a heterodimer with PcCDC50A and PcCDC50B (the expression of PcCDC50C in yeast was low, precluding further investigation) [27]. PcATP2/PcCDC50B was reported to hydrolyse ATP in the presence of PS and PE [27]. Together, these findings suggest that PfATP2 might be able to form an active complex with more than one of the PfCDC50 proteins *in vitro*, but that within the parasite it primarily associates with PfCDC50C.

It was reported that the depletion of PfCDC50C by conditional gene disruption did not impact the uptake of NBD-PS (or NBD-PE or NBD-PC) by parasitised erythrocytes (as measured after 30 min at 37°C) [52]. We observed a modest but significant effect of GlcN exposure on the overall uptake of NBD-PS by erythrocytes infected with PfATP2-HAreg parasites under similar conditions. Compared to experiments with isolated parasites at 15°C, measurements of NBD-PS uptake by parasitised erythrocytes (at 37°C) provide a less direct measure of PfATP2 activity and are more complex to interpret. For example, it is possible that endocytosis of host cell cytosol into the parasite contributes to the observed NBD-PS uptake by parasitised erythrocytes and that this is affected by PfATP2 expression level. The greater number of pathways involved in total NBD-PS uptake by parasitised erythrocytes may explain the less pronounced effect of GlcN (and MMV compounds at 2 µM) on parasitised erythrocytes compared to isolated parasites. Given the complexity of the system, we did not pursue studies with erythrocytes infected with wild-type parasites (+/- GlcN) or PfATP2-overexpressing parasites to further investigate whether the effect of GlcN on NBD-PS uptake by erythrocytes infected with PfATP2-HAreg parasites can be attributed to PfATP2-HA knockdown.

In other organisms, a range of substrates have been identified for different P4-ATPases including PS, PE, PC, glucosylceramide and galactosylceramide (reviewed in [26]). The *in situ* assay developed to measure PfATP2 activity in this study could be useful to further investigate the substrate range and transport properties of PfATP2. The assay can also be used to study the regulation of PfATP2 – for example to test whether its activity is stimulated by signalling molecules such as phosphatidylinositol-4-phosphate (PI4P) (as observed for PcATP2 [27] and also expected for PfATP2 based on the presence of a GYAFS motif [53,60]). It remains to be determined whether PS is the sole substrate of PfATP2 or whether it has additional substrates. PcATP2 was suggested to transport both PS and PE [27], whereas *T. gondii* ATP2B was found to serve as a PS flippase in the *T. gondii* plasma membrane [58,59] but was reported not to transport PE [59]. Depletion of *T. gondii* ATP2A did not affect NBD-PS uptake by the parasites (NBD-PE was not tested) [58]. Furthermore, it has been shown that NBD-PS internalisation by isolated *P. falciparum* parasites was partially inhibited by the P-type ATPase inhibitor vanadate but that NBD-PE internalisation was not [50]. In light of the challenges we encountered when attempting to extract NBD-PE from parasite membranes with BSA, further investigation of whether PE is a substrate of PfATP2 is warranted using a different approach.

Our finding that PfATP2-HA knockdown substantially reduces NBD-PS internalisation in ATP-replete (but not ATP-depleted) parasites is consistent with PfATP2 serving as a major contributor to ATP-dependent PS uptake in parasites. However, contributions by other proteins cannot be ruled out. In addition to PfATP2, the *P. falciparum* genome is predicted to encode three other putative P4-ATPases (PfATP7, PfATP8 and PfATP11) and two proteins that contain both P4-ATPase and guanylyl cyclase domains (GCα and GCβ) [5]. Only PfATP2, PfATP8 and PfGCα were predicted to be essential in asexual blood-stage parasites [31], with PfGCα subsequently confirmed to be essential (for parasite egress) [61] and PfATP2 now confirmed to be important for parasite growth in this study. The localisation of PfATP7, PfATP8 and PfATP11 in blood-stage *P. falciparum* parasites has not yet been reported. A study with the murine parasite *P. yoellii* reported that ATP7 is required for parasite survival in the mosquito and that PC is its likely substrate [62]. GCα has been reported to be expressed maximally during the schizont stage and to localise to vesicular structures in the cytoplasm [61], while GCβ is dispensible (and not expressed highly) in asexual blood-stage parasites [63].

There remains a significant level of NBD-PS internalisation in ATP-depleted parasites, and in ATP-replete parasites exposed to the P-type ATPase inhibitor vanadate, suggesting that one or more ATP-independent processes also contribute to parasite PS uptake. Scramblases are a type of transporter that catalyse bidirectional lipid transport and do not hydrolyse ATP. Blood-stage *P. falciparum* parasites have been shown to express a phospholipid scramblase (PfPLSCR); however, it appears to be localised intracellularly in trophozoite-stage parasites and it is not clear whether there is any protein present on the plasma membrane during this stage [64]. The contributor(s) to ATP-independent PS internalisation remain to be determined.

When considered together, our findings with MMV665794 and MMV007224 suggest that they are likely to target PfATP2, although additional experiments using an alternate approach are needed to determine whether the compounds directly interact with the protein. We found that parasites in which PfATP2 was knocked down became hypersensitive to growth inhibition by MMV665794 and MMV007224 (and MMV665852), and that parasites in which PfATP2 was overexpressed displayed reduced sensitivity to the compounds. This is consistent with the compounds targeting PfATP2, as when there is less of the protein, the degree to which it must be inhibited to reduce the parasite’s PfATP2 activity to a deadly level is lower. We showed that MMV665794 and MMV007224 inhibit NBD-PS internalisation by ATP-replete parasites at lower concentrations than those needed to inhibit NBD-PS uptake by ATP-depleted parasites. Nevertheless, the concentrations required to detect inhibition of NBD-PS uptake by ATP-replete parasites in our assays were higher than the IC_50_ values of the compounds in (longer-term) growth assays. Decreasing the concentration of NBD-PS five-fold to 1 µM appeared to increase the potency of the MMV compounds (cf. **Fig 6A**, **6B**, **6C and 6D**), suggesting that NBD-PS and the MMV compounds might compete for binding to PfATP2, although the EC_50_ values for the compounds remained 5–11 fold higher than the IC_50_s obtained (for PfATP2-HA Control parasites) in proliferation assays. Under physiological conditions the concentration of PfATP2 substrate(s) in the vicinity of the transporter’s substrate binding site may be lower still, which could explain the greater potency of the compounds in growth assays. Unfortunately, we were unable to determine whether MMV665852 also inhibits NBD-PS uptake at concentrations significantly higher than its IC_50_, as a result of the higher IC_50_ of this compound and its low aqueous solubility [42].

The amplification of *pfatp2* [4] or its 1.5-fold overexpression (this study) only gave rise to a modest reduction in parasite sensitivity to MMV665794, MMV007224 and MMV665852 (~ 1.5-fold increase in IC_50_). The low level of resistance may reflect the low level of PfATP2 overexpression. It is also possible that the expression of PfCDC50C could become limiting for PfATP2 activity when PfATP2 is overexpressed. It remains to be determined whether parasites can also acquire resistance to the compounds via point mutations in PfATP2 that affect their binding.

Additional detrimental effects of the compounds in parasite membranes (and perhaps also in the membrane of the host erythrocyte) may also limit the extent to which changes in *pfatp2* expression can protect parasites from the MMV compounds. All three MMV compounds are lipophilic (with LogP values between 6 and 6.7 in Pubchem; computed using XLogP3 3.0 [65]) and have been found previously to dysregulate parasite pH [10]. In this study we found that MMV665794 and MMV007224 are likely to dysregulate pH as a result of having protonophore activity. It should be noted that protonophore activity is expected to reduce both the membrane potential [66] and pH gradient across the plasma membrane (and possibly also across internal membranes including the mitochondrial membrane), with multiple detrimental flow-on effects to cell physiology (see, e.g., [67]). We found that the concentrations of the compounds that gave rise to a detectable decrease in pH_cyt_ were similar to those that gave rise to a detectable decrease in NBD-PS internalisation. It is possible that pH dysregulation becomes the primary mode of action of the compounds in parasites that overexpress PfATP2. The MMV compounds may also have additional targets in parasite (and host cell) membranes that have not yet been discovered, and it is possible that multiple effects of the compounds interact to mediate parasite killing.

P4-ATPases and the membrane asymmetry they bring about have been found to contribute to numerous processes in diverse cell types, regulating the biophysical properties of membranes and playing an important role in protein-membrane interactions, cell signalling, vesicle-mediated transport and (in some cases) lipid scavenging from a host [26,53]. In *T. gondii*, ATP2B (but not ATP2A) was found to be important for parasite growth [58]. ATP2B was found to have a particularly important role in the secretion of microneme contents [58], motility, parasite egress from host cells and invasion of new host cells, and was not required for normal intracellular growth [59]. In contrast, PfCDC50C was found to be important for the maturation of trophozoites within host erythrocytes [52], and the MMV compounds studied here inhibited parasite growth when applied for 24 h within one cycle (starting with rings), consistent with previous data [40,41]. A previous study investigating the activity of Malaria Box compounds at different stages of intraerythrocytic development determined that MMV665794, MMV007224 and MMV665852 are active against ring-stage parasites and against either trophozoites (MMV007224 and MMV665852) or schizonts (MMV665794) [41]. PfCDC50C was proposed to play a role in the endocytosis of host haemoglobin [52], suggesting that PfATP2/PfCDC50C-mediated PS asymmetry may be important for this process. *P. falciparum* is able to synthesise PS, PE and PC from precursors and to (directly or indirectly) interconvert between them [68,69]; thus, it is not clear whether scavenging PS from the host would be an essential role for PfATP2 in blood-stage parasites.

While low-level resistance of parasites to MMV007224 and MMV665852 was reported in the Cowell *et al.* study and associated with amplification of *pfatp2* [4], multiple attempts to generate resistance to MMV665794 through *in vitro* evolution experiments with wild-type parasites (3D7 and Dd2) were unsuccessful [70]. However, parasites with low-level resistance to MMV665794 (2-2.5 fold elevated IC_50_s) were subsequently generated successfully by exposing ‘hypermutator’ parasites with mutations in DNA polymerase δ to the compound [70]. Attempts to increase the level of resistance further were not successful [70]. Interestingly, parasite lines with low-level resistance to MMV665794 were found to have mutations in a gene encoding a protein of unknown function with four predicted transmembrane domains (PF3D7_1359900; ‘Quinoxaline-resistance protein 1’ (QRP1)) [70]. Parasites engineered to express the resistance-associated QRP1 mutations (D1863Y or G1612V) were found to display low level resistance to both MMV665794 and MMV007224 (MMV665852 was not tested) [70]. Results of a genome-wide transposon mutagenesis study [31], together with the finding of a frameshift mutation in the gene encoding QRP1 in MMV007224-selected parasites [70], provide strong evidence that QRP1 is not essential in blood-stage parasites, and therefore that the primary mode of action of the MMV compounds is unlikely to be inhibition of QRP1 function. Whether QRP1 has a role in regulating PfATP2 activity, or influences parasite susceptibility to MMV665794 and MMV007224 via a different mechanism, remains to be determined.

MMV665794, MMV007224 and MMV665852 all display toxicity in some assays [41] and would need to be optimised for further development. Whether the compounds’ toxicity against human cells stems from their protonophore activity, inhibition of one or more of the 14 human P4-ATPases or different mechanism(s) is not known. We observed that MMV665794 and MMV007224 inhibited NBD-PS internalisation by uninfected erythrocytes, which suggests that the compounds might have activity against one or more PS uptake pathway(s) on the erythrocyte plasma membrane. There is evidence for the presence of three P4-ATPases in human erythrocytes: ATP11A, ATP11B and ATP11C, with ATP11C being the most highly expressed [71–73].

Recently, some quinoxaline-based compounds (similar in structure to MMV665794 and MMV007224) active against *Schistosoma* parasites were found to be more potent against *P. falciparum* than the compounds tested in this study, with *pfatp2* amplification and QRP1 mutations again shown to confer low-level resistance [74]. Further studies on whether PfATP2 and its homologues in other parasites can be exploited as new drug targets are eagerly awaited. Screening compounds for their activity against the PfATP2-HA knockdown line generated here would provide an efficient starting point for pinpointing additional PfATP2 inhibitors, with measurements of NBD-PS internalisation serving as a secondary assay for any ‘hits’ identified. Should PfATP2 inhibitors with suitable properties for animal studies be identified, an important next step would be to test their efficacy in murine models of malaria, and to determine the extent to which amplification of *atp2* protects parasites from the compounds *in vivo*.

## Methods

### Ethics statement

The use of human blood in this study (from anonymous donors) was approved by the Australian National University Human Research Ethics Committee (Protocol numbers 2011/266 and 2017/351).

### Compounds

MMV007224 was purchased from Vitas-M Laboratory. MMV665794, and additional quantities of MMV007224, were kindly provided as powders by MMV. MMV665852 was purchased from Key Organics.

16:0–06:0 NBD-PS (1-palmitoyl-2-{6-[(7-nitro-2–1,3-benzoxadiazol-4-yl)amino]hexanoyl}-sn-glycero-3-phosphoserine (ammonium salt); Avanti) and 16:0–06:0 NBD-PE (1-palmitoyl-2-{6-[(7-nitro-2–1,3-benzoxadiazol-4-yl)amino]hexanoyl}-sn-glycero-3-phosphoethanolamine; Avanti) were purchased from Merck.

### Plasmid constructs

To overexpress PfATP2 (Pf3D7_1219600) in *P. falciparum* parasites, cDNA was prepared from 3D7 parasites, and the full-length cDNA encoding PfATP2 was amplified with primers P1 and P2 (S1 Table), carrying XhoI and KpnI restriction sites, respectively. A double stop codon was added to the 3’ end of the cDNA. The resulting amplicon was then cloned into the pGlux-1 vector [75], which carries a selection marker gene encoding human DHFR, using In-Fusion HD cloning (TAKARA). The *P. falciparum* chloroquine resistance transporter (*pfcrt*) promoter was used to drive *pfatp2* expression.

To engineer the PfATP2-HAreg line, a 996 bp fragment was amplified from a region of the 3D7 *pfatp2* coding sequence immediately upstream of the stop codon using primers P3 and P4, which harbour BglII and PstI restriction sites, respectively. The fragment was ligated into the BglII and PstI sites of an HA glms vector [76].

To engineer the PfATP2-GFPreg line, the same 996 bp fragment was amplified using primers P3 and P5, which contain BglII and KpnI sites, respectively. The fragment was ligated into the BglII and KpnI sites of pGFP_*glmS* [38].

Before transfection, the production of the desired constructs was confirmed by Sanger sequencing at the Genome Discovery Unit - ACRF Biomolecular Resource Facility, The John Curtin School of Medical Research, Australian National University. The primers used for sequencing are shown in S1 Table.

### Parasite culture and transfection

Blood-stage *P. falciparum* parasites (the 3D7 strain, the PfABCI3-HAreg line [39], and the transgenic parasites made in this study) were maintained within human erythrocytes (typically Group O + , 4% haematocrit) in RPMI 1640 medium containing 25 mM HEPES and GlutaMAX (ThermoFisher Scientific Cat. # 72400120), and supplemented with 11 mM (additional) glucose, 200 μM hypoxanthine, 24 μg/mL gentamicin and 6 g/L Albumax II (defined as complete medium in this study). PfABCI3-HAreg, PfATP2-HAreg, 3D7-PfATP2+ and 3D7-EV parasites were maintained in the presence of 5 nM WR99210, and PfATP2-GFPreg parasites were cultured in the presence of 5 µM blasticidin. Cultures were kept in a horizontally-rotating shaking incubator at 37°C under a low-oxygen atmosphere (1% O_2_, 3% CO_2_ and 96% N_2_). Parasites were synchronised by sorbitol treatment [77].

Transfections were performed when parasites (3D7 strain) were predominantly at the young ring stage, with 100 μg of the appropriate circular DNA constructs transfected by electroporation as described previously [78]. Transgenic parasites were selected with 5 nM WR99210 (Jacobus Pharmaceuticals; applied 48 h post-transfection) (PfATP2-HAreg, 3D7-PfATP2+ and 3D7-EV) or 5 µM blasticidin (PfATP2-GFPreg) and were cultured every second day until parasites resistant to the selection agents were observed. For PfATP2-HAreg and PfATP2-GFPreg, this was followed by two cycles each consisting of one week with the appropriate selection agent followed by one week without. The presence of integrated transgenic DNA in the PfATP2-HAreg and PfATP2-GFPreg parasites was determined by PCR from total parasite genomic DNA (extracted from isolated trophozoite-stage parasites (see below) using a QIAGEN DNeasy Plant Kit) using PrimerSTAR GXL DNA polymerase (TAKARA). All primers used for PCRs and sequencing are listed in S1 Table. Primer P30 paired with P26 (PfATP2-HAreg) or P27 (PfATP2-GFPreg) were used to investigate whether transfection was successful, and primer P8 paired with P26 (PfATP2-HAreg) or P27 (PfATP2-GFPreg) was used to check for successful integration (with P8/P29 used to detect the unmodified locus). For PfATP2-HAreg and PfATP2-GFPreg, clonal lines were obtained using the FACS-based approach described previously [79]. The presence of the desired integration events in the clones were confirmed by PCR using the primers outlined above, and by sequencing using primer P8.

### Parasite isolation from host erythrocytes

Trophozoite-stage parasites were isolated from their host erythrocytes by adding saponin (final concentration 0.05% w/v) to parasite cultures (~ 4% haematocrit, 2–10% parasitemia), inverting the tube several times to mix, then centrifuging immediately (1000 × g, 5 min). The supernatant medium was removed and the parasites were resuspended then washed several times (with 12,000 × g, 30 s centrifugation steps) in ‘bicarbonate-free medium’ (bicarbonate-free RPMI 1640 medium supplemented with 25 mM HEPES, 11 mM (additional) glucose and 200 µM hypoxanthine; pH 7.1) at 37˚C (unless stated otherwise).

### *pfatp2* mRNA quantification

Quantitative reverse transcription PCR (qRT-PCR) was performed to quantify the expression level of *pfatp2* mRNA. Total RNA was extracted from isolated trophozoite-stage parasites using the QIAGEN RNeasy mini kit. DNA-free RNA samples (1–2 μg) were reverse-transcribed using oligo dT(18) and random primers (50˚C for 1 h in 20 μL reactions) with SuperScriptIII enzyme as recommended by the manufacturer (Invitrogen). The reaction mixes were then diluted 1:3 in nuclease-free water. qPCR was performed using FastStart Universal SYBR Green Master (ROX) (Roche) in 10 μL reactions, with each reaction containing 5 μL Master mix, 2 μL of diluted cDNA template and 0.5 μM of each oligonucleotide primer (S1 Table, P6-P15 and P31-32; see S1 Data for the primers used in each experiment). The qPCR was performed with the ViiA 7 Real-Time PCR System (ThermoFisher Scientific). The 2^-∆∆Ct^ method [80] was used to calculate relative changes in gene expression.

### Monitoring parasite viability by flow cytometry

The effect of PfATP2-HA knockdown on the survival of PfATP2-HAreg parasites was assessed using a method similar to that described previously [81]. On Day 0 (at which time parasites were in the trophozoite stage), parasitemia was accurately determined using a previously described flow cytometry method [79]. After determining the starting parasitemia, two 50 mL cultures were set up with PfATP2-HAreg parasites at a parasitemia of 0.5%. GlcN (5 mM) was added to one culture, while an equivalent volume (0.5 mL) of solvent (culture medium without Albumax II) was added to the other. WR99210 (5 nM) was present in both cultures. Every second day (for 10 days), the parasitemia was measured by flow cytometry, and the parasitised erythrocytes in both cultures were diluted by the same factor with uninfected erythrocytes (with the dilution yielding a 0.5% parasitemia for the culture lacking GlcN).

Comparisons of the growth of 3D7-EV and 3D7-PfATP2 + were performed in a similar way, except that the parasitemia was adjusted to 1% for both parasite lines and the parasitemia measured again after one cycle.

### Western blots

Western blots were used to determine whether PfATP2-HA was successfully knocked down in PfATP2-HAreg parasites exposed to GlcN. Trophozoite-stage parasites were isolated from cultures that had been maintained with GlcN (5 mM for 7 days) or without GlcN. Parasite pellets (obtained by centrifuging the parasites at 12,000 x g for ~ 30 s and removing the supernatant solution) were frozen at -80˚C then thawed. The following were then added to the (~ 60 µL) sample: 40 µL 25 mM MgCl_2_, 1 µL 100x Protease Inhibitor Cocktail (Set III) (Merck Millipore), and 1 µL of Pierce Universal Nuclease for Cell Lysis (250 units; ThermoFisher Scientific). The sample was vortexed then heated at 37°C for 5 min. NuPAGE LDS Sample Buffer (1 × ; 100 µL) and 1 × NuPAGE Sample Reducing Agent (40 µL) were then added, followed by 1 × PBS to bring the sample volume up to 400 µL. The samples were run on NuPAGE Bis-Tris Mini Protein Gels (4–12%). After completion of electrophoresis, proteins in the gel were transferred to a nitrocellulose membrane following the NuPAGE Technical Guide (ThermoFisher Scientific). The membrane was then incubated in blocking buffer (4% w/v skim milk in 1 × PBS) overnight at 4°C with constant shaking. The membranes were then exposed to specific primary and secondary antibodies (diluted in blocking buffer) for 1.5 h at room temperature in a humidified container. The primary antibodies used in this study were an anti-HA rat monoclonal antibody (1:500 dilution, clone 3F10, Roche) and an anti-heat shock protein 70 (HSP70) mouse monoclonal antibody (1:2000 dilution, a kind gift from Prof. Alex Maier). The secondary antibodies used were an anti-rat goat horseradish peroxidase (HRP)-conjugated antibody (1:5000 dilution, ab97057, Abcam) or an anti-mouse goat HRP-conjugated antibody (1:5000 dilution, ab6789, Abcam). Subsequently, the membranes were washed with 1 × PBS and visualised using Pierce ECL Plus Western Blotting Substrate with a ChemiDoc MP Imaging System (Bio-Rad).

### Localisation of PfATP2-GFP in live cells

Erythrocytes infected with trophozoite-stage *P. falciparum* parasites (PfATP2-GFPreg) were centrifuged (2,000 × *g*, 30 s) and resuspended in pH 7.4 Physiological Saline (125 mM NaCl, 5 mM KCl, 1 mM MgCl_2_, 20 mM glucose, 25 mM HEPES; pH 7.4) containing 20 μg/mL Hoechst 33258. After a 15 min incubation at 37˚C, the cells were washed twice then resuspended in pH 7.4 Physiological Saline. An aliquot of the cell suspension was then added to the centre of a microscope slide, covered with a coverslip and sealed with nail polish. For the images shown in **Fig 1**, the cells were observed with an Applied Precision DeltaVision Elite system (GE Healthcare) with an inverted IX71 microscope with a 100X UPlanSApo oil immersion lens (Olympus). Images were taken using a Photometrics Cool SNAP HQ2 camera and deconvolved and adjusted for contrast and brightness using SoftWoRx Suite 2.0 software. GFP fluorescence was detected using wavelengths of 490 nm (excitation) and 525 nm (emission). Hoechst 33258 was detected at 350 nm (excitation) and 455 nm (emission). Images were processed using ImageJ software. For the image of an erythrocyte infected with a ring-stage PfATP2-GFPreg parasite (S2 Fig), a Leica STELLARIS 8 confocal microscope with a HC PL APO CS2 63x/1.40 OIL objective was used at the Centre for Advanced Microscopy (Australian National University). Images were taken using Leica Application Suite X (LAS-X) software. GFP fluorescence was detected using wavelengths of 488 nm (excitation) and 495–650 nm (emission), Hoechst 33258 with wavelengths of 405 nm (excitation) and 419–480 nm (emission), and transmitted light with Differential Interference Contrast (DIC). Images were processed using LAS-X Office software.

### Immunofluorescence assays

Immunofluorescence assays (IFAs) were performed with infected erythrocytes according to a published method [82]. In brief, parasitised erythrocytes (on some occasions separated from uninfected erythrocytes using a Miltenyi Biotec VarioMACS magnet) were fixed with 4% paraformaldehyde and 0.0075% glutaraldehyde in 1 × PBS for 30 min. After washing with 1 × PBS, cells were permeabilised in 0.1% Triton X-100 in 1 × PBS for 10 min, treated with 0.1 M glycine in H_2_O for 15 min, then incubated overnight in 3% BSA in 1 × PBS. Anti-HA High Affinity (1:200 dilution; clone 3F10, Roche) and rabbit anti-EXP2 antibody (1:500 dilution; [83]) were used for the primary antibody. After a 1 h incubation with primary antibody, cells were washed three times in 1 × PBS and then incubated with Alexa Fluor 488 conjugated Donkey anti-Rat IgG (H + L) Highly Cross-Adsorbed Secondary Antibody (1:500 dilution; Catalog # A-21208, ThermoFisher Scientific) or Alexa Fluor 546 conjugated Goat anti-Rabbit IgG (H + L) Highly Cross-Adsorbed Secondary Antibody (1:400 dilution; Catalog # A-11035, ThermoFisher Scientific) for 1 h. The cells were treated with DAPI (Molecular Probes) for 5 min before being mounted on a slide. Microscopy was performed using an Applied Precision DeltaVision Elite system (as above) and images were processed using ImageJ.

### Measurements of parasite pH_cyt_

Parasite pH_cyt_ was measured under physiological conditions and in the presence of a low external [Cl^-^] using methods similar to those described previously [44,48]. Briefly, isolated trophozoite-stage parasites suspended in bicarbonate-free medium were loaded with the pH-sensitive fluorescent dye BCECF by incubating them with BCECF-AM (5 µM) for 10 min at 37˚C.

In the experiments for which data are shown in **Fig 3**, the parasites were then washed twice in pH 7.1 Physiological Saline (125 mM NaCl, 5 mM KCl, 1 mM MgCl_2_, 20 mM glucose, 25 mM HEPES; pH 7.1) (with 12,000 × *g* 30 s centrifugation steps) and resuspended in this solution. The parasites were added to the compounds of interest (or DMSO alone) diluted in pH 7.1 Physiological Saline in wells of a 96-well plate and fluorescence was recorded immediately.

In the experiments for which data are shown in **Fig 4**, the parasites were washed and resuspended in Glucose-free Saline (135 mM NaCl, 5 mM KCl, 1 mM MgCl_2_, 25 mM HEPES; pH 7.1) after dye loading, and incubated at 37˚C for 20 min to allow for the depletion of ATP. The parasite suspension (15 µL per well) was then added to the compounds of interest (or DMSO alone) diluted in Glucose-free Cl^-^-free Saline (135 mM Na^+^-gluconate, 5 mM K^+^-gluconate, 1 mM MgSO_4_, 25 mM HEPES (pH 7.1)), yielding a final volume per well of 200 µL and an external [Cl^-^] of 11 mM, and fluorescence was recorded immediately.

For all pH_cyt_ experiments, fluorescence was measured at 37˚C with excitation wavelengths of 440 nm (yielding pH-insensitive fluorescence) and 495 nm (yielding pH-sensitive fluorescence). Emission was recorded at 520 nm.

To convert Fluorescence Ratios (495 nm/440 nm) into pH_cyt_ values, calibrations were included in all pH_cyt_ experiments. Parasites were suspended (at a density matching that used in the experimental wells) in calibration salines of known pH (130 mM KCl, 1 mM MgCl_2_, 20 mM glucose, 25 mM HEPES; pH 6.8, 7.1, 7.4, 7.8) containing nigericin (a compound that facilitates H^+^/K^+^ exchange; final concentration 5 µM), with fluorescence recorded for 5 min. The average of the Fluorescence Ratio values obtained during the read were plotted against the pH of the calibration solution, and a line was fit to the data. The resulting equation was used to convert Fluorescence Ratio values to pH_cyt_.

### Measurements of parasite volume

Measurements of the volume distribution of saponin-isolated trophozoites (washed and resuspended in pH 7.1 Physiological Saline at 37˚C) were carried out using a Beckman Coulter Multisizer 4 fitted with a 100 μm aperture tube [84]. The electrolyte solution within the aperture tube was pH 7.1 Physiological Saline. The mean volume of the parasites within each sample was determined by fitting a Gaussian distribution curve to the population data within a 4–120 fL window.

### Parasite proliferation assays

To evaluate the effects of compounds on the *in vitro* proliferation of parasites, a SYBR Safe-based fluorescence assay was used [85,86]. The starting parasitemia was 1% (with predominantly ring-stage parasites), the haematocrit was 1%, and the duration of the assay was 72 h. *P. falciparum*-infected erythrocytes incubated in the presence of 0.5 μM chloroquine were used as a non-proliferation control and those incubated in the absence of any compounds served as a 100% parasite proliferation control. The DMSO concentration in the assays did not exceed 0.1% (v/v). Parasite proliferation was measured and calculated as described previously [86]. The parasite proliferation data were fitted with the equation Y = Bottom + (Top – Bottom)/(1 + (IC_50_/X)^Hillslope) using GraphPad Prism, where Y represents percentage parasite proliferation, IC_50_ represents the concentration of the test compound resulting in 50% inhibition of parasite proliferation, and X represents the compound concentration.

In the experiments for which data are shown in S3 Fig, the compounds were washed off 24 h into the 72 h experiment. Two washes were performed (with 1000 × g, 5 min centrifugation steps), bringing the concentration of compound in each well to ~ 3% of its starting concentration.

### Phospholipid internalisation assays

In all measurements of NBD-PS and NBD-PE uptake performed in this study, the final concentration of DMSO (used as a solvent for the MMV compounds) was 0.1% v/v and the concentration of water (solvent for vanadate) was 2% v/v. The concentration of ethanol (solvent for NBD-PS and NBD-PE) was 0.1% v/v in experiments performed with 1 µM NBD-PS (the experiments performed with parasitised erythrocytes, as well as the experiments with isolated parasites for which data are shown in **Fig 6C–E**) and 0.5% v/v in experiments performed with 5 µM NBD-PS or NBD-PE (all other experiments).

For most experiments, phospholipid internalisation assays were performed with saponin-isolated trophozoite-stage parasites. After isolation, the parasites were washed three times in pH 7.1 Physiological Saline (37˚C). In the case of parasites that had been exposed to 5 mM GlcN in the lead up to the experiments, 5 mM GlcN was present for the first two washes but not included in the third wash (and was absent for the remainder of the experiment). Parasites that were not exposed to GlcN were exposed to an equivalent volume of the solvent (complete medium lacking Albumax II; final concentration 1% v/v) during the relevant steps. For measurements performed with ATP-depleted parasites, parasites were washed and resuspended in Glucose-free Saline (containing 5 mM GlcN where appropriate), then incubated for ~ 15 min at 37˚C. The parasites were washed in Glucose-free Saline to remove GlcN prior to the NBD-PS uptake measurements. With the exception of the experiments for which data are shown in **Figs 6C–E**, S8 and S12A (in which parasites were maintained at 37˚C prior to, and during, the NBD-PS uptake measurements where indicated), all other measurements with isolated parasites were carried out at 15˚C. Parasites (90 µL) were added to 100 µL of pH 7.1 Physiological Saline (or Glucose-free Saline for measurements with ATP-depleted parasites) (pre-warmed to 15˚C) containing the compound of interest (or solvent alone). Unless stated otherwise, parasites were exposed to test compounds for 10 min while cooling to 15˚C, before the addition of 10 µL NBD-PS (final concentration 5 µM). Similar results were obtained when compounds were added to parasites at the same time as NBD-PS (S14 Fig). To terminate NBD-phospholipid uptake at the desired time point, an aliquot of cell suspension (150 µL) was transferred to a tube containing 1 mL ice-cold pH 7.1 Physiological Saline (or Glucose-free Saline for measurements with ATP-depleted parasites) containing 4% w/v fatty-acid free BSA. The samples were centrifuged (12,000 × g, 1 min, 4˚C), the supernatant solution discarded, and the parasites resuspended in 1 mL of the same BSA-containing solution. The samples were centrifuged again, the supernatant solution discarded, and the parasites resuspended in 600 µL pH 7.1 Physiological Saline (or Glucose-free Saline in the case of ATP-depleted parasites) (37˚C) containing the DNA stain Hoechst 33258 (2 µg/mL) for 15 min. The parasites were then washed and resuspended in 300 µL pH 7.1 Physiological Saline and maintained at room temperature until they were analysed by flow cytometry using a BD LSRII cytometer at the Cytometry, Histology and Spatial Multiomics Facility (Australian National University). The excitation wavelength was 488 nm for NBD-phospholipid fluorescence (530/30 nm emission filter), and 405 nm for Hoechst 33258 fluorescence (450/50 emission filter). Twenty thousand cells were sampled at low sampling speed, typically with the following settings: forward scatter = 532 V (log scale), side scatter = 205 V (log scale), FITC = 852 V (log scale) and Pacific Blue = 418 V (log scale). The gating strategy is shown in S15 Fig. The geometric mean of NBD-phospholipid fluorescence for the population in the Hoechst 33258 positive, NBD-phospholipid positive gate was determined with FlowJo.

For experiments with erythrocytes (parasitised and uninfected) we adopted an approach described previously [52]. Erythrocytes (5–10% of which were infected with trophozoite-stage parasites) were washed and resuspended in bicarbonate-free medium and exposed to the test compounds of interest for 10 min at 37˚C. NBD-PS (final concentration 1 µM) and Hoechst 33258 (4 µg/mL) were then added and cells (final haematocrit 3.5-4.5%) were incubated at 37˚C for 30 min. The cells were then centrifuged (12,000 × g ~ 30 s) and washed twice with 1 mL bicarbonate-free medium containing 5% w/v fatty-acid free BSA (37˚C). The cells were then resuspended in 1 mL 1 × PBS and analysed by flow cytometry (as above).

### Microscopic determination of NBD-PS localisation

The location of NBD-PS was investigated in saponin-isolated trophozoite-stage parasites that had been exposed to 5 μM NBD-PS at 15°C in the dark for 9 min then washed with ice-cold 4% w/v BSA in pH 7.1 Physiological Saline (as above) then resuspended in pH 7.1 Physiological Saline. Imaging was performed at the Centre for Advanced Microscopy (Australian National University) on a Leica STELLARIS 8, using a HC PL APO CS2 63x/1.40 OIL objective, with the Leica Lightning adaptive deconvolution module in the Leica Application Suite X (LAS-X) software (version 4.7.0.28176). The final fluorescent images were obtained using the wavelengths of 467 nm (excitation wavelength), 484 nm to 732 nm (emission wavelength), and transmitted light with Differential Interference Contrast (DIC).

## Supporting information

S1 Table Primers used in this study. Restriction enzyme sites are underlined.
(PDF)

S1 Fig Integration of the PfATP2-HA-glmS and PfATP2-GFP-glmS targeting constructs into the endogenous *pfatp2* locus. (**A**,**B**) Schematics showing the strategy used to incorporate the HA (**A**)/GFP (**B**) and glmS ribozyme sequences at the 3’ end of the *pfatp2* gene by single crossover homologous recombination. UTR, untranslated region; HA, 3x Haemagglutinin epitope tag; glmS, glmS ribozyme sequence; hDHFR, human dihydrofolate reductase (selectable marker); bsd, blasticidin deaminase (selectable marker). (**C**) Image of PCR products run on an agarose gel. Primers P8 and P26 (PfATP2-HAreg) or P8 and P27 (PfATP2-GFPreg) were used to test for integration of the desired DNA (expected sizes ~1.6 kb and ~1.5 kb, respectively) and primers P8 and P29 were used to test for the presence of the unmodified *pfatp2* gene (expected size ~1.66 kb). Lanes 1 and 5: PCRs performed without sample DNA (no template control; NTC). Lanes 2 and 6: PCRs performed with wild-type 3D7 parasites. Lane 3: PCRs performed with PfATP2-HAreg before cloning. Lane 4: PCRs performed with PfATP2-HAreg clone F11 (the clone used throughout the study). Lane 7: PCRs performed with PfATP2-GFPreg before cloning. Lane 8: PCRs performed with PfATP2-GFPreg clone 2F5 (for which data are shown in S2 Fig). Lane 9: PCRs performed with PfATP2-GFPreg clone 1F2 (for which data are shown in Fig 1A). The lane labelled M was loaded with Hyperladder 1 kb (Bioline).
(TIF)

S2 Fig PfATP2-GFP is expressed in ring-stage parasites. Image of an erythrocyte infected with a ring-stage PfATP2-GFPreg parasite (-GlcN; 2F5 clone) showing the presence of DNA (Hoechst 33258) and PfATP2-GFP. Scale bar = 5 µm.
(TIF)

S3 Fig Growth inhibition of PfATP2-HA knockdown and Control parasites after a 24 h exposure to MMV665794 (A), MMV007224 (B) and MMV665852 (C). The data for parasites in which PfATP2-HA was knocked down (PfATP2-HAreg + GlcN) are shown in pink and the data for PfATP2-HAreg parasites that were not exposed to GlcN (-GlcN) are shown in blue. Where present, GlcN (5 mM) was added to cultures four days before the start of the experiments and maintained throughout the experiments. The duration of the assays was 72 h, with the compounds washed off 24 h after the start of the assay (see Methods). The data are from three independent experiments performed on different days, except for the highest and lowest concentrations, for which data are n = 1–2. The IC_50_ values (mean ± SEM, n = 3) were 82 ± 25 nM (-GlcN) and 34.5 ± 10.0 nM (+GlcN) for MMV665794 (*P* = 0.03, ratio paired t-test); 220 ± 12 nM (-GlcN) and 90 ± 21 nM (+GlcN) for MMV007224 (*P* = 0.05, ratio paired t-test); and 852 ± 183 nM (-GlcN) and 343 ± 70 nM (+GlcN) for MMV665852 (*P* = 0.002, ratio paired t-test).
(TIF)

S4 Fig The BSA extraction strategy we employed to study NBD-PS uptake was not suitable for studies with NBD-PE. Isolated 3D7 trophozoites were exposed to 5 µM NBD-PE at 15˚C. At each time point, parasites were transferred to (then washed in) an ice-cold solution either containing BSA (as described for NBD-PS experiments in Methods) or not containing BSA. **A** shows the NBD-PE fluorescence (geometric mean) (mean ± range/2 of technical duplicates) for the parasites that were exposed to the BSA extraction procedure (black) or not exposed to BSA (red). In **B**, the mean values for the + BSA condition from the same experiment are expressed as a percentage of those for the -BSA condition. The data are from a single experiment, and are representative of similar experiments that also showed that NBD-PE fluorescence in parasites did not increase significantly with NBD-PE exposure time, and that a high proportion of NBD-PE appeared to be non-extractable even at early time points.
(TIF)

S5 Fig Flow cytometry analysis of fluorescent NBD-PS uptake in isolated trophozoites. (**A**-**F**) NBD-PS internalisation was measured in isolated trophozoite-stage parasites suspended in pH 7.1 Physiological Saline at 15˚C. The parasites were incubated with either a compound (**D**-**F**) or solvent alone (0.1% v/v DMSO; **A**-**C**) for 10 min, then NBD-PS (5 µM) uptake was measured over 9 min. NBD-PS internalisation was measured in: (**A**) *pfatp2*-overexpressing 3D7-PfATP2+ parasites (red) and empty vector control parasites (3D7-EV; blue); (**B**) PfATP2-HAreg parasites in which PfATP2-HA was knocked down (red; two day exposure of culture to 5 mM GlcN) or expressed at a normal level (-GlcN; blue); (**C**) wild-type 3D7 parasites from cultures that were either exposed to 5 mM GlcN for two days in the lead-up to the experiment (red) or that were not exposed to GlcN (blue); (**D**-**F**) PfATP2-HAreg Control parasites (-GlcN) in the absence (blue) or presence (red) of (**D**) 500 µM vanadate, (**E**) 10 µM MMV665794 (**F**) 15 µM MMV007224. (**G**-**I**) NBD-PS internalisation was measured in isolated trophozoite-stage PfATP2-HAreg parasites suspended in pH 7.1 Physiological Saline at 37˚C. The parasites were incubated with either a compound (**H**,**I**) or solvent alone (**G**) for 10 min, then NBD-PS (1 µM) uptake was measured over 30 min. (**G**) PfATP2-HA Control (-GlcN; blue) and knockdown (red; two day exposure of culture to 5 mM GlcN) parasites exposed to solvent alone (0.1% v/v DMSO). (**H**,**I**) PfATP2-HAreg Control parasites (-GlcN) in the absence (blue) or presence (red) of (**H**) 2 µM MMV665794 or (**I**) 2 µM MMV007224. All panels: cells were gated for Hoechst 33258 fluorescence and NBD-PS fluorescence using the strategy shown in S15 Fig. In each panel, data from a single experiment, representative of at least three independent experiments, are shown.
(TIF)

S6 Fig Comparisons of the growth and volume of 3D7-EV and 3D7-PfATP2+ parasites. (**A**) The parasitemia of 3D7-EV, 3D7-PfATP2+ and (wild-type) 3D7 cultures measured one cycle (~ 48 h) after adjusting the parasitemia of both cultures to 1%. The bars and error bars show the mean ± SEM from seven independent experiments (all performed on different days) for 3D7-EV and 3D7-PfATP2+ cultures and five independent experiments for wild-type 3D7 cultures. The symbols show the data from each individual experiment. The *P* value is from a lognormal one-way ANOVA in which the data for the three parasite strains were compared. (**B**) The mean volume of saponin-isolated 3D7-EV and 3D7-PfATP2+ trophozoites. The bars and error bars show the mean ± SEM from four independent experiments, each performed on different days, with 3D7-EV and 3D7-PfATP2 + parasites tested at the same time. The symbols show the mean volume of the parasite populations measured in each individual experiment. The *P* value is from a ratio paired t-test. One of the volume measurements was performed on the same day as an NBD-PS uptake assay. On that day the mean volume of 3D7-PfATP2+ parasites (31 fL) was similar to that of 3D7-EV parasites (32 fL) yet the former internalised NBD-PS at a greater rate (1.4-fold greater slope).
(TIF)

S7 Fig The effects of exposing PfATP2-HAreg or PfABCI3-HAreg parasites to GlcN for one or two days on parasite volume and NBD-PS internalisation. (**A**,**C**) The mean volumes of isolated trophozoite-stage PfATP2-HAreg (**A**) and PfABCI3-HAreg (**C**) parasites from cultures that had been exposed to 5 mM GlcN for one day or two days in the lead up to the experiment, expressed as a percentage of those obtained for the equivalent parasites that had not been exposed to GlcN. The *P* values are the results of ratio paired t-tests (with comparisons made to the -GlcN Control) performed with the pre-normalised data (volume in fL). The mean volumes measured in the different experiments ranged from 19-59 fL. (**B**,**D**) NBD-PS internalisation (measured over 9 min at 15˚C) by isolated trophozoite-stage PfATP2-HAreg (**B**) or PfABCI3-HAreg (**D**) parasites that had been exposed to 5 mM GlcN for one day or two days, expressed as a percentage of that observed for the equivalent parasites that had not been exposed to GlcN. The *P* values show the results of ratio paired t-tests performed on pre-normalised data (NBD-PS fluorescence (geometric mean), which ranged from 2183-24602 in different experiments). The Day 2 data in panel **B** summarise data from all the different experiments (n = 31) performed in this study in which NBD-PS internalisation was measured for 9 min at 15˚C (in the presence of solvent alone) in isolated ATP-replete PfATP2-HAreg parasites from cultures that were either not exposed to GlcN or exposed to 5 mM GlcN for two days, drawing together data from Figs 5B, 5E, 6A, 6B, S8B, S10A, S10B, S11B, S11C and S14. In all panels, the bars and error bars show the mean and SEM, and the symbols show the data from individual biological replicates (performed on different days). *P* values indicating statistical significance (< 0.05) are shown in bold. GlcN was not present during the measurements.
(TIF)

S8 Fig The effects of PfATP2-HA knockdown and vanadate on NBD-PS uptake by isolated trophozoite-stage parasites at 37˚C. Both panels show data for PfATP2-HA knockdown (pink; from cultures exposed to 5 mM GlcN for two days) and Control (blue; -GlcN) parasites. GlcN was not present during the measurements. (**A**) The parasites were suspended in pH 7.1 Physiological Saline at 37˚C, with NBD-PS uptake measured at the time points indicated. The *P* value obtained from a ratio paired t-test performed with the slopes obtained when lines were fit to the pre-normalised data (NBD-PS fluorescence (geometric mean)) is shown. (**B**) The parasites were suspended at pH 7.1 Physiological Saline at either 37˚C or 15˚C, in the absence (solvent control) or presence of vanadate (500 µM), with NBD-PS internalisation measured at the 9 min time point. In **A**, the data shown are the mean ± SEM; in **B**, the symbols show the data from individual experiments, and the bars and error bars show the mean ± SEM. In both panels, the data are from four (**A**) or three (**B**) independent experiments (performed on different days; with all conditions and parasite types tested concurrently) and are expressed as a percentage of the NBD-PS fluorescence (geometric mean) measured under the conditions indicated on the y axis. In **B**, the NBD-PS fluorescence (geometric mean) was higher at 37˚C (ranging from 19784-55878 for the solvent control -GlcN parasites in the different experiments) than at 15˚C (range: 5021–11144). The numbers above the bars are *P* values from paired t-tests performed on the normalised data, with significant values (*P* < 0.05) indicated in bold.
(TIF)

S9 Fig The effects of GlcN and vanadate (500 µM) on NBD-PS uptake by erythrocytes infected with PfATP2-HAreg parasites (A) and uninfected erythrocytes (B). NBD-PS uptake was measured over 30 min in cells suspended in bicarbonate-free medium at 37˚C. The cells were either exposed to 5 mM GlcN for two days in the lead up to the experiment to reduce PfATP2-HA expression (+GlcN; pink) or not exposed to GlcN (-GlcN; blue). GlcN was not present during the NBD-PS uptake measurements. The bars and error bars show the mean ± SEM (from four independent experiments performed on different days, with all conditions tested concurrently) and the symbols show the data from individual experiments. The data are expressed as a percentage of the NBD-PS fluorescence (geometric mean) measured in the -GlcN Control. The NBD-PS fluorescence for the -GlcN Control (geometric mean) was higher in parasitised erythrocytes (ranging from 2406 in experiment 1 (lowest) to 52323 in experiment 3 (highest)) than in uninfected erythrocytes (ranging from 795 in experiment 1–9143 in experiment 3). Statistical comparisons were made using ratio paired t-tests on the pre-normalised data (NBD-PS fluorescence (geometric mean)). The *P* values are shown, with values indicating statistical significance (*P* < 0.05) shown in bold. Coloured values are the *P* values for comparisons with the solvent control for the same cell type.
(TIF)

S10 Fig CCCP did not inhibit the internalisation of NBD-PS by Control or PfATP2-HA knockdown parasites when tested at 100 nM (A) or 5 µM (B). NBD-PS internalisation by isolated trophozoite-stage parasites was measured at 15˚C over 9 min. Symbols show data from each individual experiment with Control parasites (PfATP2-HAreg -GlcN; blue) and PfATP2-HA knockdown parasites (exposed to 5 mM GlcN for two days; pink). GlcN was not present during the NBD-PS uptake measurements. The bars and error bars show the mean ± SEM from four (**A**) or three (**B**) independent experiments (each performed on different days). The data are expressed as a percentage of the NBD-PS fluorescence (geometric mean) observed in Control parasites that were not treated with CCCP (0.1% v/v DMSO; solvent control). The *P* values from ratio paired t-tests performed with the pre-normalised data are shown (with significant values (*P* < 0.05) shown in bold).
(TIF)

S11 Fig MMV665794 and MMV007224 inhibit NBD-PS internalisation by ATP-replete parasites at lower concentrations than those needed to affect ATP-depleted parasites. NBD-PS internalisation was measured over 9 min in isolated trophozoite-stage parasites (ATP-replete parasites suspended in pH 7.1 Physiological Saline or ATP-depleted parasites suspended in Glucose-free Saline) at 15˚C, in the absence (0.1% v/v DMSO; solvent control) or presence of the compounds and concentrations indicated. The experiments were performed with PfATP2-HA knockdown parasites (pink; from cultures exposed to 5 mM GlcN for two days) and Control parasites (blue; PfATP2-HAreg -GlcN). GlcN was not present when parasites were exposed to NBD-PS. The data are expressed as a percentage of the NBD-PS fluorescence (geometric mean) measured in the ATP-replete parasites that were not exposed to GlcN or test compound. The data are from three (**A**,**B**) or four (**C**) independent experiments, in which all the conditions/parasite types shown within one panel were tested concurrently. The symbols show the data from individual experiments; the bars and error bars show the mean ± SEM. The solvent control data in **A** are the same as three of those shown in Fig 5E (as vanadate and the MMV compounds were tested together in three experiments). The data shown in the Fig 6 insets were derived from the same experiments as the data shown in this Figure (but in the case of the Fig 6 insets were normalised to the data for ATP-depleted parasites that were not exposed to GlcN or test compound).
(TIF)

S12 Fig The effects of 10 µM MMV665794 and 15 µM MMV007224 on NBD-PS uptake by isolated trophozoite-stage parasites expressing different levels of PfATP2 at 15°C and 37˚C. (**A**) PfATP2-HA knockdown (pink; from cultures exposed to 5 mM GlcN for two days) and Control (blue; PfATP2-HAreg -GlcN) parasites were suspended in pH 7.1 Physiological Saline at either 37˚C or 15˚C, in the absence (solvent control) or presence of 10 µM MMV665794 or 15 µM MMV007224, with NBD-PS internalisation measured at the 9 min time point. As noted in S8B Fig, the NBD-PS fluorescence (geometric mean) was higher at 37˚C than at 15˚C. A three-way ANOVA performed with the pre-normalised data revealed a significant effect of the MMV compounds (*P* = 0.003), temperature (*P* = 0.004) and PfATP2-HA knockdown (*P* = 0.05) on NBD-PS uptake, but no significant effect of temperature on the effects of the MMV compounds (*P* = 0.07) or PfATP2-HA knockdown (*P* = 0.7). (**B**,**C**) NBD-PS uptake (9 min, 15°C) in 3D7-PfATP2+ (light blue) and 3D7-EV (purple) parasites (**B**) and 3D7 parasites (+GlcN (grey) or -GlcN (teal); **C**) in the absence (solvent control) or presence of 10 µM MMV665794 or 15 µM MMV007224. The *P* values for comparisons with the solvent control for the same parasite type, from lognormal one-way ANOVAs with post hoc Dunnett’s tests performed on the pre-normalised data, are shown (with significant values (*P* < 0.05) indicated in bold). In all panels, the data are from three independent experiments (performed on different days; with all conditions tested concurrently) and are expressed as a percentage of the NBD-PS fluorescence (geometric mean) measured under the conditions indicated on the y axis. The symbols show the data from individual experiments, and the bars and error bars show the mean ± SEM. GlcN was not present during the measurements. The MMV compounds were tested at the same time as vanadate; thus the control data shown in panels **A**, **B** and **C** are the same as those shown in Figs 5A (inset), S8B and 5D (inset), respectively.
(TIF)

S13 Fig The effects of GlcN, MMV665794 and MMV007224 on NBD-PS uptake by erythrocytes infected with PfATP2-HAreg parasites (A,C,E) and uninfected erythrocytes (B,D,F). NBD-PS uptake was measured over 30 min in cells suspended in bicarbonate-free medium at 37˚C. The cells were either exposed to 5 mM GlcN for two days in the lead up to the experiment to reduce PfATP2-HA expression (+GlcN; pink) or not exposed to GlcN (-GlcN; blue). GlcN was not present during the NBD-PS uptake measurements. The bars and error bars show the mean ± SEM (from four (**A**,**B**) or three (**C**-**F**) independent experiments performed on different days (with all conditions tested concurrently) and the symbols show the data from individual experiments. The data are expressed as a percentage of the NBD-PS fluorescence (geometric mean) measured in the -GlcN Control. The NBD-PS fluorescence for the -GlcN Control (geometric mean) was higher in parasitised erythrocytes (ranging from 2406 (lowest) to 52323 (highest)) than in uninfected erythrocytes (ranging from 795 (lowest) to 9646 (highest)). In **A** and **B**, the MMV compounds were tested in the same experiments as vanadate; thus, the Control data are the same as those shown in S9 Fig. The experiments for which data are shown in **C** and **D** were performed 5.0-5.5 h earlier than those for which data are shown in **E** and **F**. In two of the three experiments, parasites were isolated from a sample of the cultures (-GlcN and +GlcN); for the later measurements (**E**), the mean parasite volumes had increased to 130–138% of those measured at the earlier timepoint (**C**). The *P* values in black are the results of ratio paired t-tests performed with the pre-normalised data (NBD-PS fluorescence (geometric mean)) (with bold indicating statistical significance (*P* < 0.05)). The data obtained for each cell type in the presence of MMV compounds was compared to the solvent control data using lognormal one-way ANOVAs with post hoc Dunnett’s tests; ns, not significant (*P* > 0.5), *P < 0.05, **P < 0.01, ***P < 0.001.
(TIF)

S14 Fig Effect of vanadate (500 µM), MMV665794 (10 µM) and MMV007224 (15 µM) on NBD-PS internalisation, with and without a 10 min preincubation, in PfATP2-HA knockdown and Control parasites. NBD-PS internalisation was measured over 9 min at 15˚C in isolated trophozoite-stage parasites suspended in pH 7.1 Physiological Saline. The cells were either exposed to 5 mM GlcN for two days in the lead up to the experiment to reduce PfATP2-HA expression (+GlcN; pink) or not exposed to GlcN (-GlcN; blue). GlcN was not present during the measurements. The bars and error bars show the mean ± SEM (from three independent experiments performed on different days, with all conditions tested concurrently) and the symbols show the data from individual experiments. The data are expressed as a percentage of the NBD-PS fluorescence (geometric mean) measured in the -GlcN Control. To determine whether preincubation had a significant effect on the activity of any of the compounds, two-way ANOVAs were performed for each compound using the normalised data. Preincubation did not significantly affect the activity of vanadate (*P* = 0.2), MMV665794 (*P* = 0.09) or MMV007224 (*P* = 0.3). In two experiments, 5 µM CCCP was tested alongside other compounds for which data are shown in this Figure. Thus, the Control data for two of the experiments in this Figure (preincubation condition) are the same as those shown in S10 Fig.
(TIF)

S15 Fig Gating strategy used to quantify the internalisation of NBD-PS (or NBD-PE) by flow cytometry. RBCs (**A**; uninfected and infected with *P. falciparum*) or isolated parasites (PfATP2-HAreg -GlcN; **B**-**D**) were first gated in a plot of SSC‐A versus FSC-A to exclude debris. Cells were then gated in plots of FSC‐W versus FSC‐H and SSC‐W versus SSC‐H, in order to exclude doublets and aggregates. The cells were then gated in a plot of Hoechst 33258 fluorescence and NBD-PS fluorescence. The data in each panel are from a single experiment, representative of three or more independent experiments. (**A**) Gating strategy for uRBCs and iRBCs (infected with PfATP2-HAreg parasites) (-GlcN, 0.1% v/v DMSO condition). (**B**-**C**) Gating strategy for isolated trophozoite-stage parasites (PfATP2-HAreg -GlcN) that were not exposed to NBD-PS (**B**) or that were exposed to NBD-PS for 9 min at 15˚ (**C**). Except for the NBD-PE experiment for which data are shown in S4 Fig (for which a similar strategy to that shown for ‘All NBD PS’ in **D** was used), the gating strategy shown in panel **C** was used throughout this study. However, forming a larger gate encompassing parasites with varying Hoechst 33258 fluorescence levels (which are expected to be more diverse in level of maturity, with some parasites likely having multiple nuclei) yielded similar results (**D**). In the bar graph, the results obtained using the smaller gate shown on the left (‘Single_NBD PS’) are shown in blue, and those obtained using the larger gate shown on the right (‘All_NBD PS’) are shown in red. The data are for ATP-replete PfATP2-HAreg Control parasites (-GlcN) that were exposed to DMSO (0.1%; solvent control), 15 µM MMV007224, 10 µM MMV665794 or 500 µM vanadate (data from the same experiments are also shown in Figs 5E and S11A). The bars and error bars show the mean + SEM from three independent experiments.
(TIF)

S1 Data Tabulated data from the study. (XLSX)
