# Supplementary material for: PfATP2 is a flippase on the Plasmodium falciparum surface that is important for growth and influences parasite sensitivity to antiplasmodial compounds
Source: PLoS Pathog. 2025 Oct 27;21(10):e1013645. doi: 10.1371/journal.ppat.1013645 (PMC12588512; doi:10.1371/journal.ppat.1013645)
Supplement: S1 Table — Restriction enzyme sites are underlined. (PDF) [file ppat.1013645.s001.pdf]

Primer name in study	Sequence (5' – 3')	Use
P1	TTACATATAACTCGAGATGTCCTTAGTTTATAGAAAAACACTGAAC	Generation of 3D7-PfATP2+
P2	TCTTCTCCTTTACTGGTACCTTATTAAATCATATTATCTTGTTTTCTAATTAATTGTATA	Generation of 3D7-PfATP2+
P3	CGAGATCTACCTGTTGTAATTCATGCTGTGT	Generation of PfATP2-HAreg and PfATP2-GFPreg, sequencing for 3D7-PfATP2+ and PfATP2-HAreg vector
P4	CGCTGCAGAATCATATTATCTTGTTTTCTAATTAATTGTATACATG	Generation of PfATP2-HAreg
P5	CGGGTACCAATCATATTATCTTGTTTTCTAATTAATTGTATACATG	Generation of PfATP2-GFPreg
P6	ATAATGAAGGGCGCACACCA	qRT-PCR and sequencing of *pfatp2* overexpression vector
P7	TCTACATCGGTTCGTTTGGCT	qRT-PCR and sequencing of *pfatp2* overexpression vector
P8	GGTGATGGAGCAAATGACCG	qRT-PCR, sequencing of *pfatp2* overexpression vector and integration checks (PfATP2-HAreg and PfATP2-GFPreg)
P9	GCGGAATTGACTAATACCATAATCTGA	qRT-PCR and sequencing of *pfatp2* overexpression vector
P10	GCTGACTACGTCCCTGCCC	qRT-PCR (18S rRNA)
P11	ACAATTCATCATATCTTTCAATCGGTA	qRT-PCR (18S rRNA)
P12	GGCAACAACAGGTCTGTGAT	qRT-PCR (18S rRNA)
P13	TTCGGCGGAGGAAAAGTATG	qRT-PCR (18S rRNA)
P14	AAGTAGCAGGTCATCGTGGTT	qRT-PCR (stRNA)
P15	AGTTCGGCACATTCTTCCATAA	qRT-PCR (stRNA)
P16	TTCAGAGGTCTCTATAG AATGTGTTATGGTATTGTAAGTGAAGAAG	sequencing for 3D7-PfATP2+
P17	AGCGTGGGTCTCGTACT ATGGTTCCTAAAAGATGAATACTTCCTAC	sequencing for 3D7-PfATP2+
P18	TGGGTAGTTATTACAGGAAATTTCGT	sequencing for 3D7-PfATP2+
P19	TCACCAAATGAAGGAACATCTGC	sequencing for 3D7-PfATP2+
P20	AAAATCGATTTAGATATTTGCCTT	sequencing for 3D7-PfATP2+
P21	TTCATGGTTGGTTCCACAGG	sequencing for 3D7-PfATP2+
P22	TCCGTTAATAATAAATACACGCAGTC	sequencing for 3D7-PfATP2+
P23	TGTGCCCATTAACATCACCATC	sequencing for 3D7-PfATP2+
P24	CAGGAAACAGCTATGAC	sequencing for PfATP2-HAreg vector
P25	TATTTAGGTGACACTATAG	sequencing for PfATP2-HAreg vector
P26	GTTTGAAGAAATCCTTACGGCTGTG	sequencing for PfATP2-HAreg vector; transfection and integration checks (PfATP2-HAreg)
P27	GTAAGTTTTCCGTATGTTGCATCACC	sequencing for pGFP_*glmS* vector; transfection and integration checks (PfATP2-GFPreg)
P28	ATTATATTTTTTTTCTTCCCACATTTCGT	sequencing for pGFP_*glmS* vector
P29	TCAATCCATAGGATATATGATATGTAAGT	Paired with P8 to detect unmodified *pfatp2* locus
P30	GATCGCGGCCGCTAACCATACGACATTTGGACCGT	Checks for successful transfection (PfATP2-HAreg and PfATP2-GFPreg)
P31	TGGGTAGTTATTACAGGAAATTTCGT	qRT-PCR
P32	TCACCAAATGAAGGAACATCTGC	qRT-PCR
