# Supplementary figures and images for: PfATP2 is a flippase on the Plasmodium falciparum surface that is important for growth and influences parasite sensitivity to antiplasmodial compounds
Source: PLoS Pathog. 2025 Oct 27;21(10):e1013645. doi: 10.1371/journal.ppat.1013645 (PMC12588512; doi:10.1371/journal.ppat.1013645)

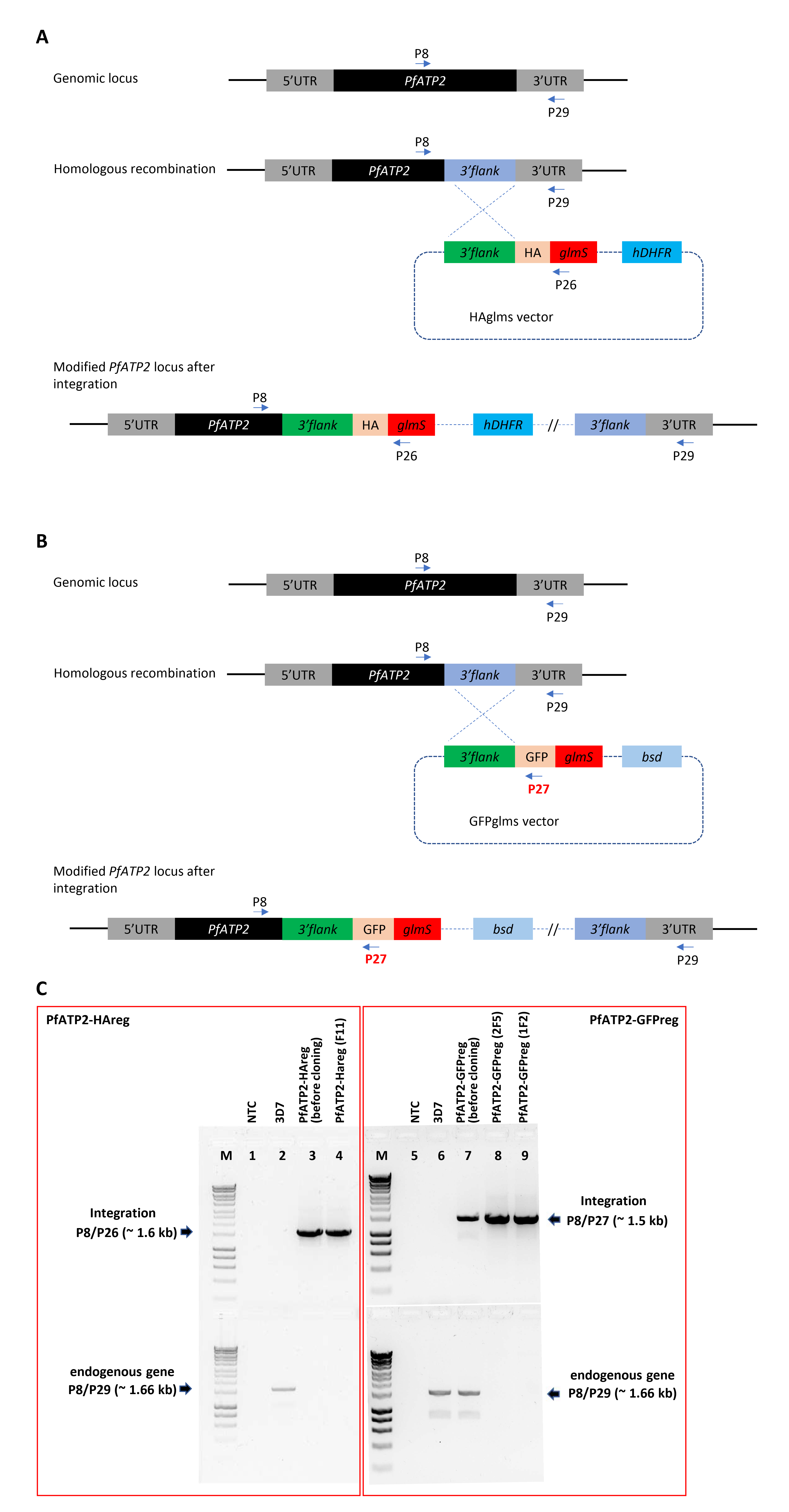

Supplement: S1 Fig — (A,B) Schematics showing the strategy used to incorporate the HA (A)/GFP (B) and glmS ribozyme sequences at the 3’ end of the pfatp2 gene by single crossover homologous recombination. UTR, untranslated region; HA, 3x Haemagglutinin epitope tag; glmS, glmS ribozyme sequence; hDHFR, human dihydrofolate reductase (selectable marker); bsd, blasticidin deaminase (selectable marker). (C) Image of PCR products run on an agarose gel. Primers P8 and P26 (PfATP2-HAreg) or P8 and P27 (PfATP2-GFPreg) were used to test for integration of the desired DNA (expected sizes ~1.6 kb and ~1.5 kb, respectively) and primers P8 and P29 were used to test for the presence of the unmodified pfatp2 gene (expected size ~1.66 kb). Lanes 1 and 5: PCRs performed without sample DNA (no template control; NTC). Lanes 2 and 6: PCRs performed with wild-type 3D7 parasites. Lane 3: PCRs performed with PfATP2-HAreg before cloning. Lane 4: PCRs performed with PfATP2-HAreg clone F11 (the clone used throughout the study). Lane 7: PCRs performed with PfATP2-GFPreg before cloning. Lane 8: PCRs performed with PfATP2-GFPreg clone 2F5 (for which data are shown in S2 Fig). Lane 9: PCRs performed with PfATP2-GFPreg clone 1F2 (for which data are shown in Fig 1A). The lane labelled M was loaded with Hyperladder 1 kb (Bioline). (TIF) [file ppat.1013645.s002.tif]

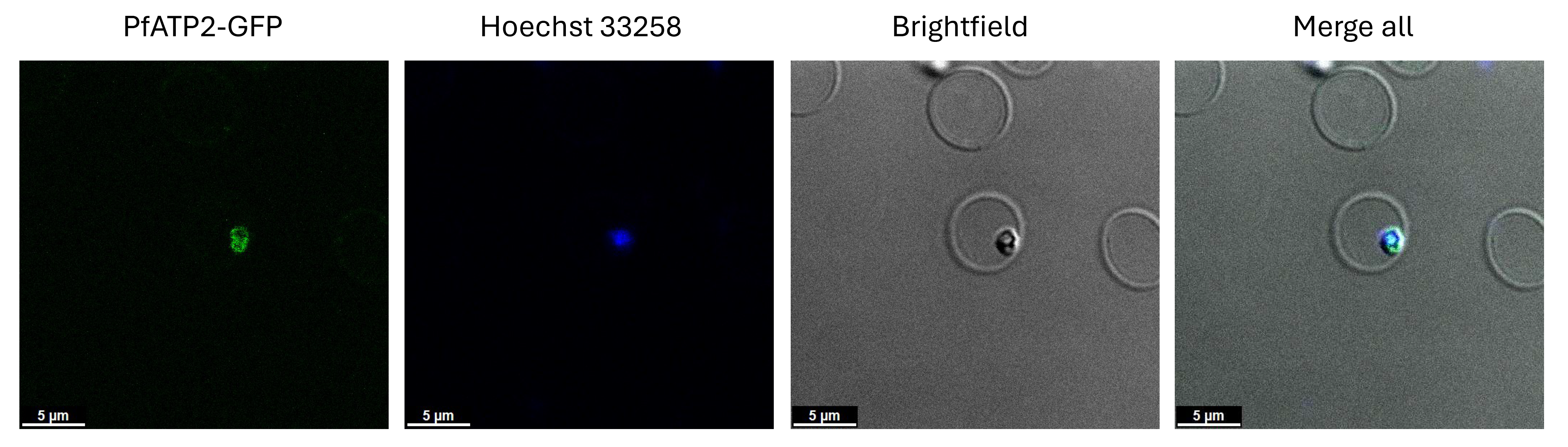

Supplement: S2 Fig — Image of an erythrocyte infected with a ring-stage PfATP2-GFPreg parasite (-GlcN; 2F5 clone) showing the presence of DNA (Hoechst 33258) and PfATP2-GFP. Scale bar = 5 µm. (TIF) [file ppat.1013645.s003.tif]

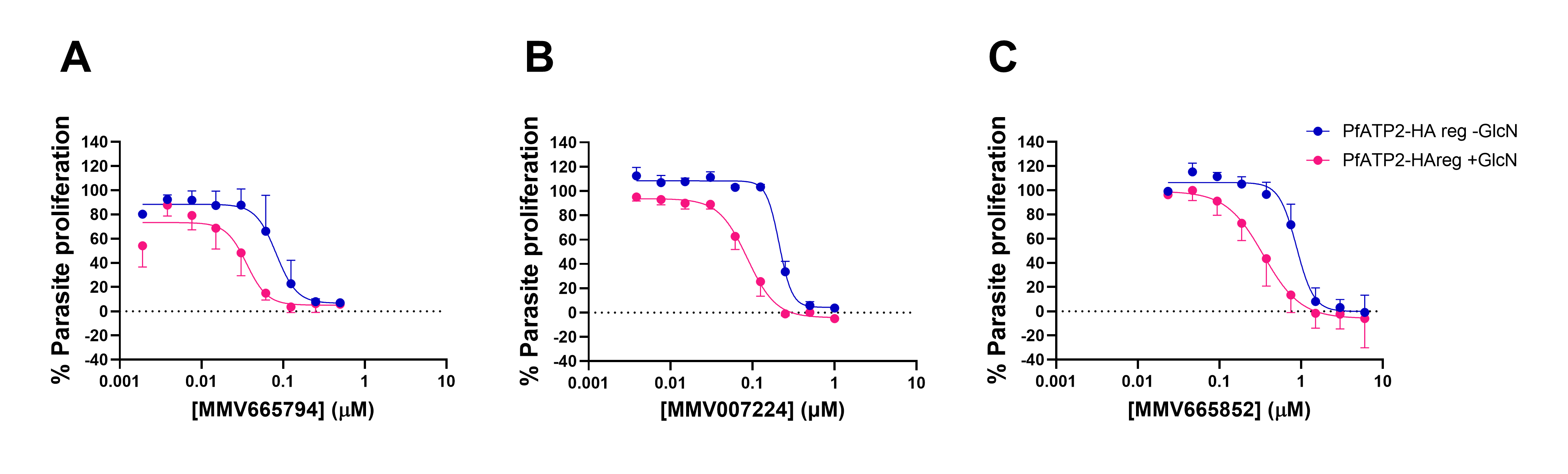

Supplement: S3 Fig — The data for parasites in which PfATP2-HA was knocked down (PfATP2-HAreg + GlcN) are shown in pink and the data for PfATP2-HAreg parasites that were not exposed to GlcN (-GlcN) are shown in blue. Where present, GlcN (5 mM) was added to cultures four days before the start of the experiments and maintained throughout the experiments. The duration of the assays was 72 h, with the compounds washed off 24 h after the start of the assay (see Methods). The data are from three independent experiments performed on different days, except for the highest and lowest concentrations, for which data are n = 1–2. The IC50 values (mean ± SEM, n = 3) were 82 ± 25 nM (-GlcN) and 34.5 ± 10.0 nM (+GlcN) for MMV665794 (P = 0.03, ratio paired t-test); 220 ± 12 nM (-GlcN) and 90 ± 21 nM (+GlcN) for MMV007224 (P = 0.05, ratio paired t-test); and 852 ± 183 nM (-GlcN) and 343 ± 70 nM (+GlcN) for MMV665852 (P = 0.002, ratio paired t-test). (TIF) [file ppat.1013645.s004.tif]

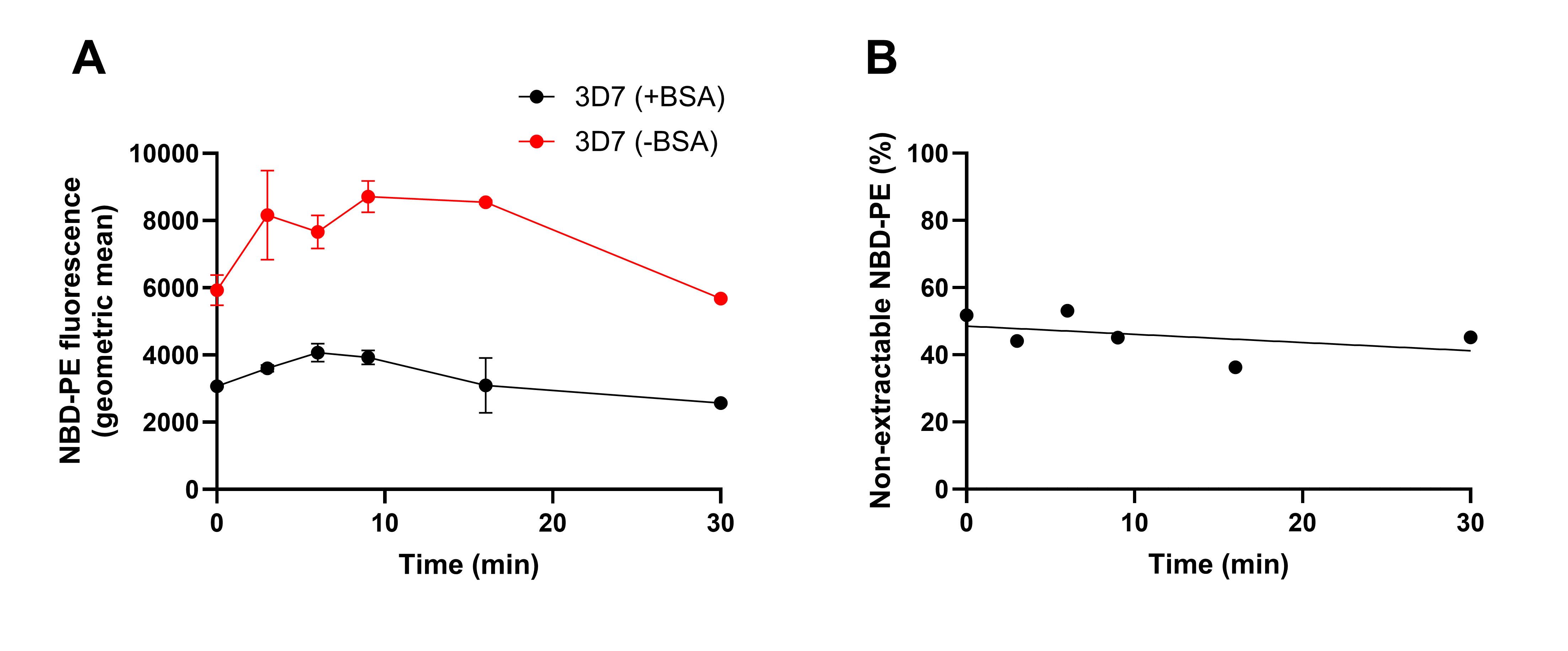

Supplement: S4 Fig — Isolated 3D7 trophozoites were exposed to 5 µM NBD-PE at 15˚C. At each time point, parasites were transferred to (then washed in) an ice-cold solution either containing BSA (as described for NBD-PS experiments in Methods) or not containing BSA. A shows the NBD-PE fluorescence (geometric mean) (mean ± range/2 of technical duplicates) for the parasites that were exposed to the BSA extraction procedure (black) or not exposed to BSA (red). In B, the mean values for the + BSA condition from the same experiment are expressed as a percentage of those for the -BSA condition. The data are from a single experiment, and are representative of similar experiments that also showed that NBD-PE fluorescence in parasites did not increase significantly with NBD-PE exposure time, and that a high proportion of NBD-PE appeared to be non-extractable even at early time points. (TIF) [file ppat.1013645.s005.tif]

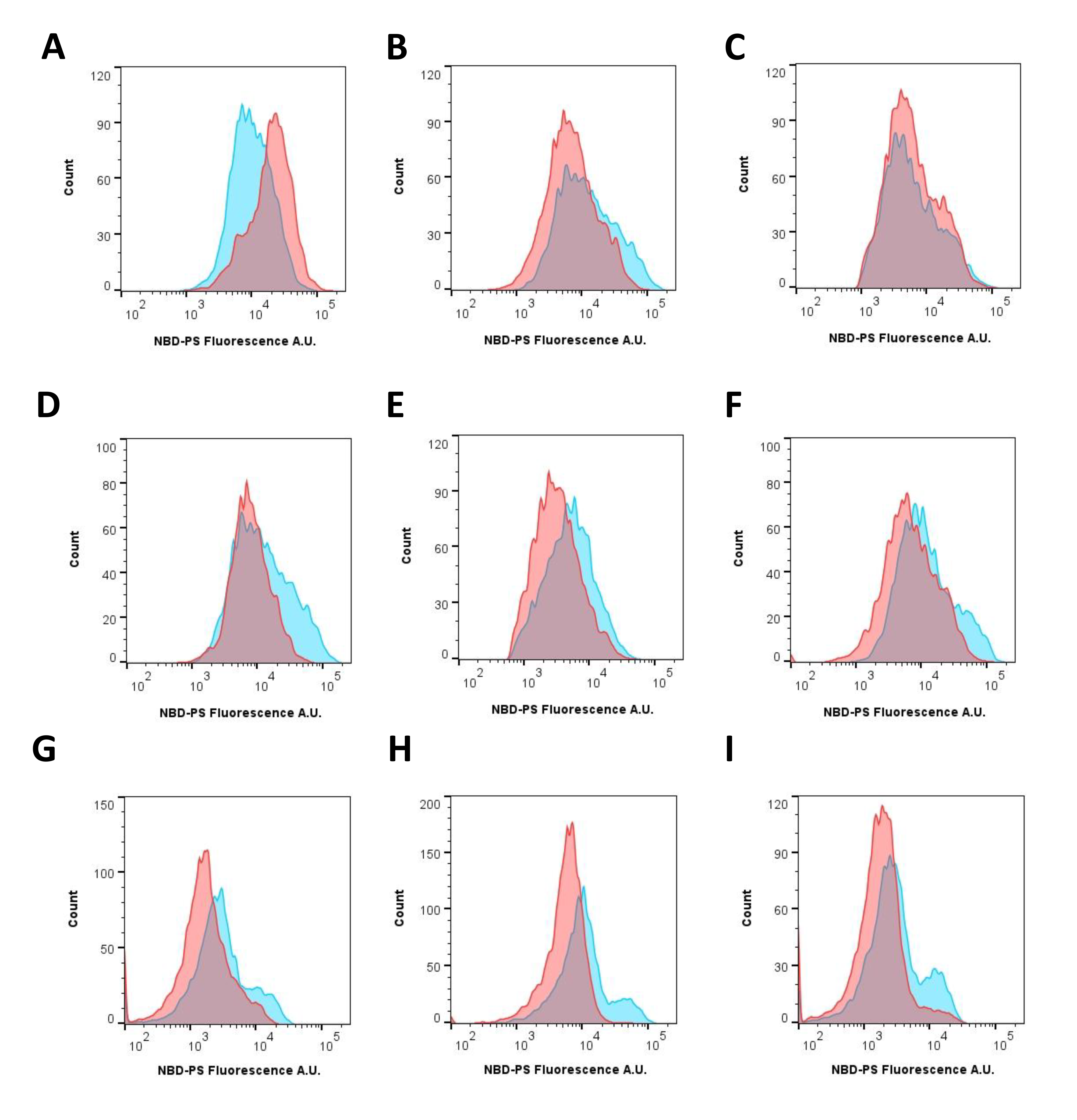

Supplement: S5 Fig — (A-F) NBD-PS internalisation was measured in isolated trophozoite-stage parasites suspended in pH 7.1 Physiological Saline at 15˚C. The parasites were incubated with either a compound (D-F) or solvent alone (0.1% v/v DMSO; A-C) for 10 min, then NBD-PS (5 µM) uptake was measured over 9 min. NBD-PS internalisation was measured in: (A) pfatp2-overexpressing 3D7-PfATP2+ parasites (red) and empty vector control parasites (3D7-EV; blue); (B) PfATP2-HAreg parasites in which PfATP2-HA was knocked down (red; two day exposure of culture to 5 mM GlcN) or expressed at a normal level (-GlcN; blue); (C) wild-type 3D7 parasites from cultures that were either exposed to 5 mM GlcN for two days in the lead-up to the experiment (red) or that were not exposed to GlcN (blue); (D-F) PfATP2-HAreg Control parasites (-GlcN) in the absence (blue) or presence (red) of (D) 500 µM vanadate, (E) 10 µM MMV665794 (F) 15 µM MMV007224. (G-I) NBD-PS internalisation was measured in isolated trophozoite-stage PfATP2-HAreg parasites suspended in pH 7.1 Physiological Saline at 37˚C. The parasites were incubated with either a compound (H,I) or solvent alone (G) for 10 min, then NBD-PS (1 µM) uptake was measured over 30 min. (G) PfATP2-HA Control (-GlcN; blue) and knockdown (red; two day exposure of culture to 5 mM GlcN) parasites exposed to solvent alone (0.1% v/v DMSO). (H,I) PfATP2-HAreg Control parasites (-GlcN) in the absence (blue) or presence (red) of (H) 2 µM MMV665794 or (I) 2 µM MMV007224. All panels: cells were gated for Hoechst 33258 fluorescence and NBD-PS fluorescence using the strategy shown in S15 Fig. In each panel, data from a single experiment, representative of at least three independent experiments, are shown. (TIF) [file ppat.1013645.s006.tif]

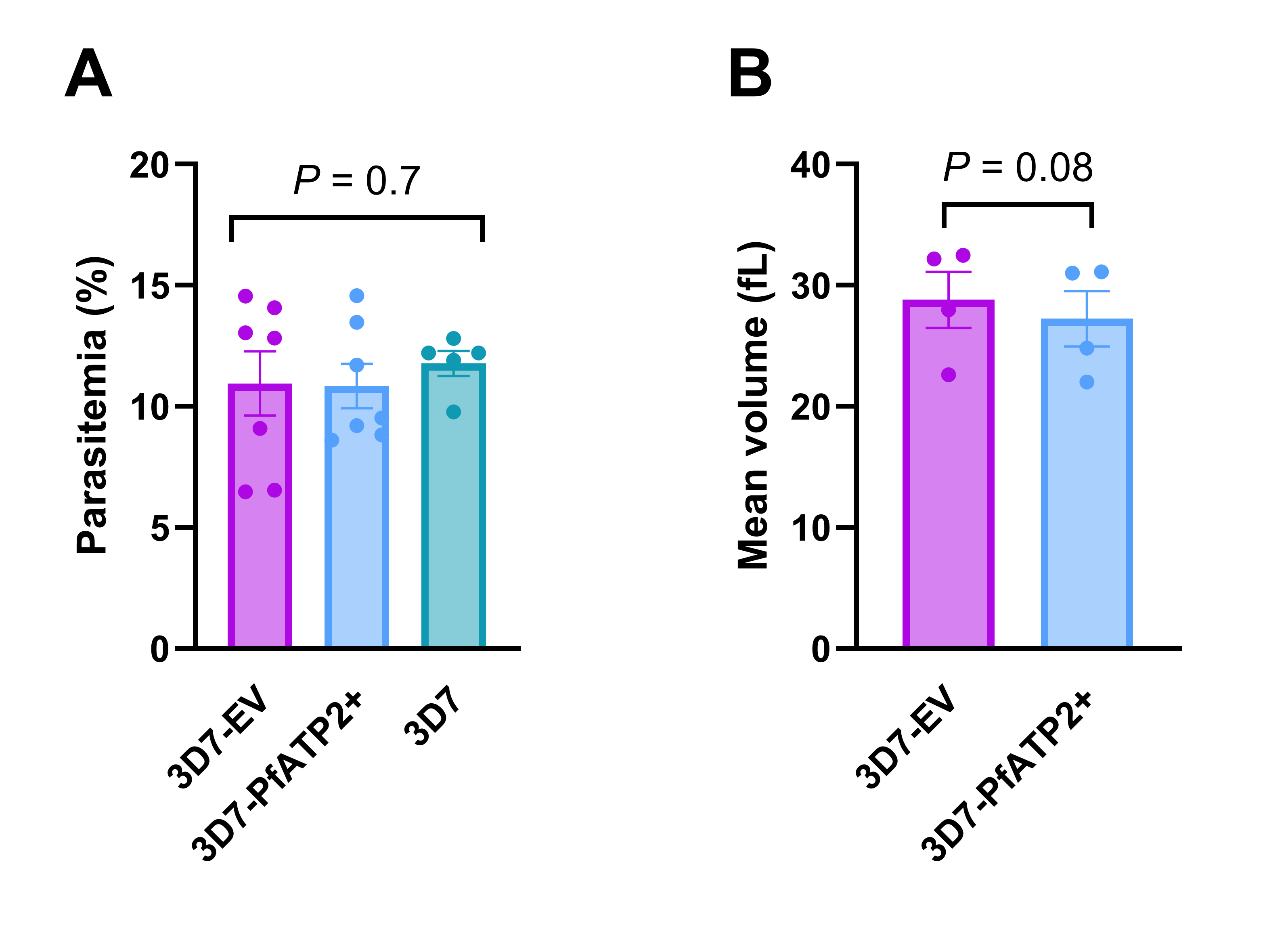

Supplement: S6 Fig — (A) The parasitemia of 3D7-EV, 3D7-PfATP2+ and (wild-type) 3D7 cultures measured one cycle (~ 48 h) after adjusting the parasitemia of both cultures to 1%. The bars and error bars show the mean ± SEM from seven independent experiments (all performed on different days) for 3D7-EV and 3D7-PfATP2+ cultures and five independent experiments for wild-type 3D7 cultures. The symbols show the data from each individual experiment. The P value is from a lognormal one-way ANOVA in which the data for the three parasite strains were compared. (B) The mean volume of saponin-isolated 3D7-EV and 3D7-PfATP2+ trophozoites. The bars and error bars show the mean ± SEM from four independent experiments, each performed on different days, with 3D7-EV and 3D7-PfATP2 + parasites tested at the same time. The symbols show the mean volume of the parasite populations measured in each individual experiment. The P value is from a ratio paired t-test. One of the volume measurements was performed on the same day as an NBD-PS uptake assay. On that day the mean volume of 3D7-PfATP2+ parasites (31 fL) was similar to that of 3D7-EV parasites (32 fL) yet the former internalised NBD-PS at a greater rate (1.4-fold greater slope). (TIF) [file ppat.1013645.s007.tif]

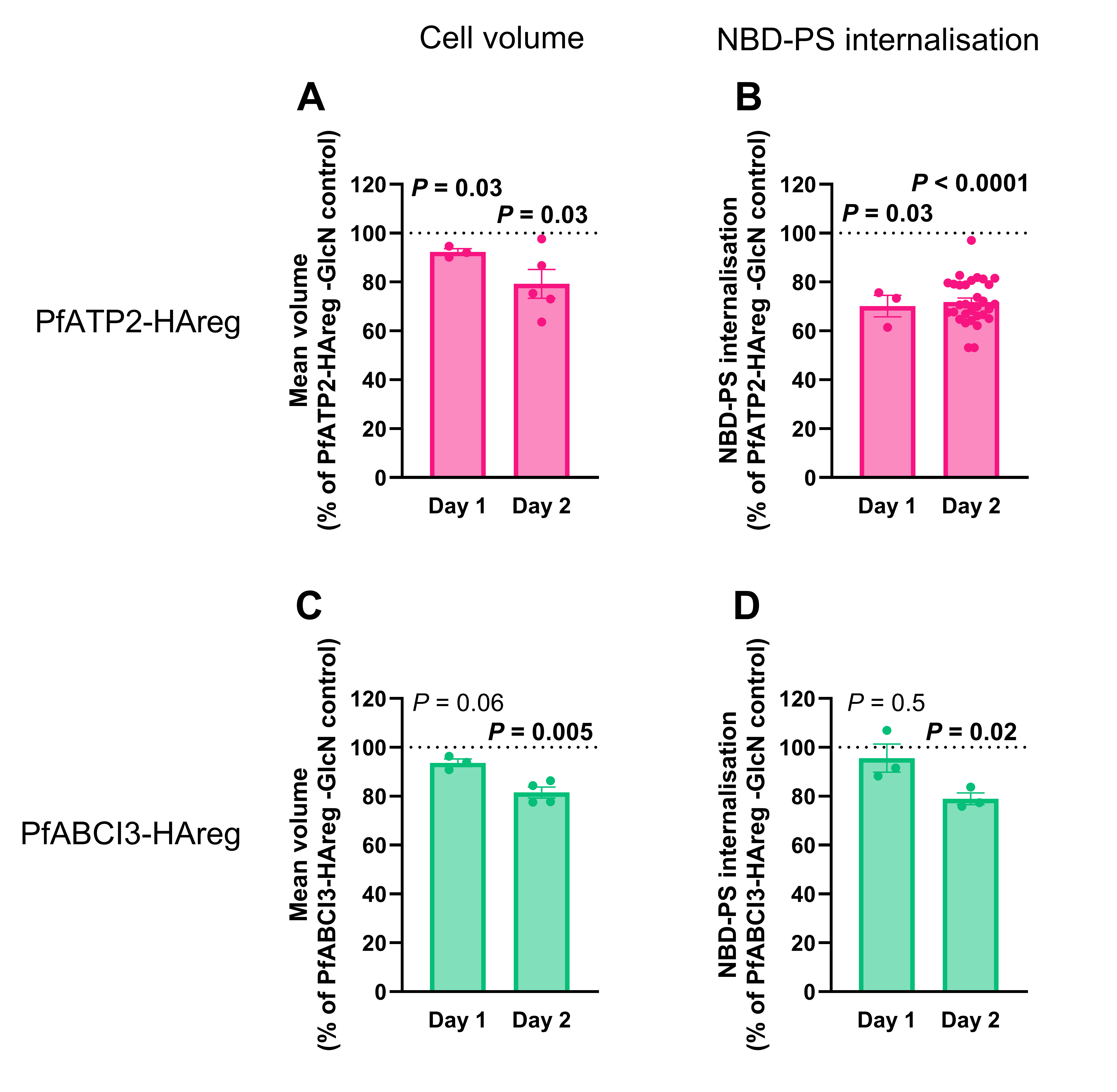

Supplement: S7 Fig — (A,C) The mean volumes of isolated trophozoite-stage PfATP2-HAreg (A) and PfABCI3-HAreg (C) parasites from cultures that had been exposed to 5 mM GlcN for one day or two days in the lead up to the experiment, expressed as a percentage of those obtained for the equivalent parasites that had not been exposed to GlcN. The P values are the results of ratio paired t-tests (with comparisons made to the -GlcN Control) performed with the pre-normalised data (volume in fL). The mean volumes measured in the different experiments ranged from 19-59 fL. (B,D) NBD-PS internalisation (measured over 9 min at 15˚C) by isolated trophozoite-stage PfATP2-HAreg (B) or PfABCI3-HAreg (D) parasites that had been exposed to 5 mM GlcN for one day or two days, expressed as a percentage of that observed for the equivalent parasites that had not been exposed to GlcN. The P values show the results of ratio paired t-tests performed on pre-normalised data (NBD-PS fluorescence (geometric mean), which ranged from 2183-24602 in different experiments). The Day 2 data in panel B summarise data from all the different experiments (n = 31) performed in this study in which NBD-PS internalisation was measured for 9 min at 15˚C (in the presence of solvent alone) in isolated ATP-replete PfATP2-HAreg parasites from cultures that were either not exposed to GlcN or exposed to 5 mM GlcN for two days, drawing together data from Figs 5B, 5E, 6A, 6B, S8B, S10A, S10B, S11B, S11C and S14. In all panels, the bars and error bars show the mean and SEM, and the symbols show the data from individual biological replicates (performed on different days). P values indicating statistical significance (< 0.05) are shown in bold. GlcN was not present during the measurements. (TIF) [file ppat.1013645.s008.tif]

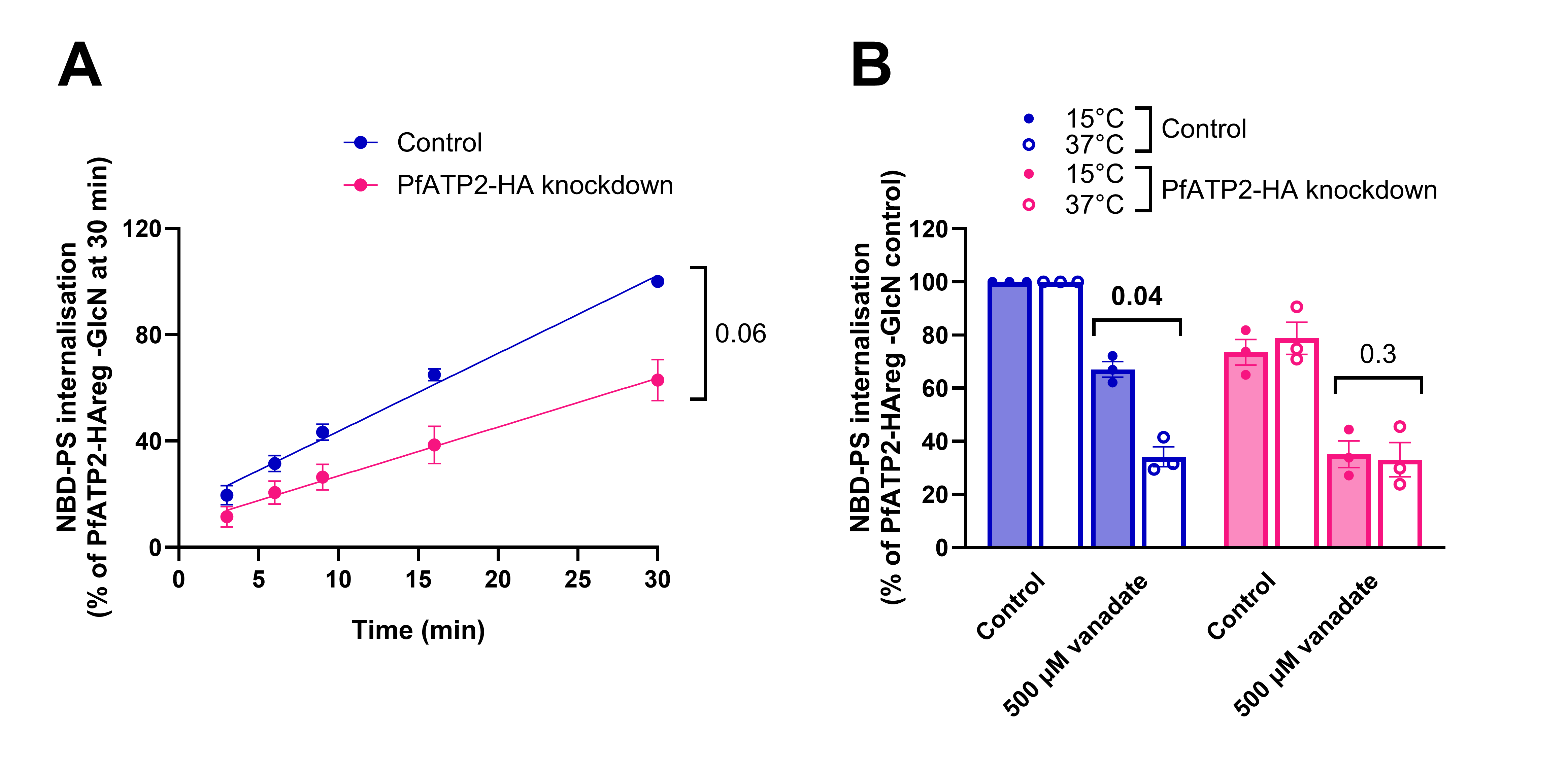

Supplement: S8 Fig — Both panels show data for PfATP2-HA knockdown (pink; from cultures exposed to 5 mM GlcN for two days) and Control (blue; -GlcN) parasites. GlcN was not present during the measurements. (A) The parasites were suspended in pH 7.1 Physiological Saline at 37˚C, with NBD-PS uptake measured at the time points indicated. The P value obtained from a ratio paired t-test performed with the slopes obtained when lines were fit to the pre-normalised data (NBD-PS fluorescence (geometric mean)) is shown. (B) The parasites were suspended at pH 7.1 Physiological Saline at either 37˚C or 15˚C, in the absence (solvent control) or presence of vanadate (500 µM), with NBD-PS internalisation measured at the 9 min time point. In A, the data shown are the mean ± SEM; in B, the symbols show the data from individual experiments, and the bars and error bars show the mean ± SEM. In both panels, the data are from four (A) or three (B) independent experiments (performed on different days; with all conditions and parasite types tested concurrently) and are expressed as a percentage of the NBD-PS fluorescence (geometric mean) measured under the conditions indicated on the y axis. In B, the NBD-PS fluorescence (geometric mean) was higher at 37˚C (ranging from 19784-55878 for the solvent control -GlcN parasites in the different experiments) than at 15˚C (range: 5021–11144). The numbers above the bars are P values from paired t-tests performed on the normalised data, with significant values (P < 0.05) indicated in bold. (TIF) [file ppat.1013645.s009.tif]

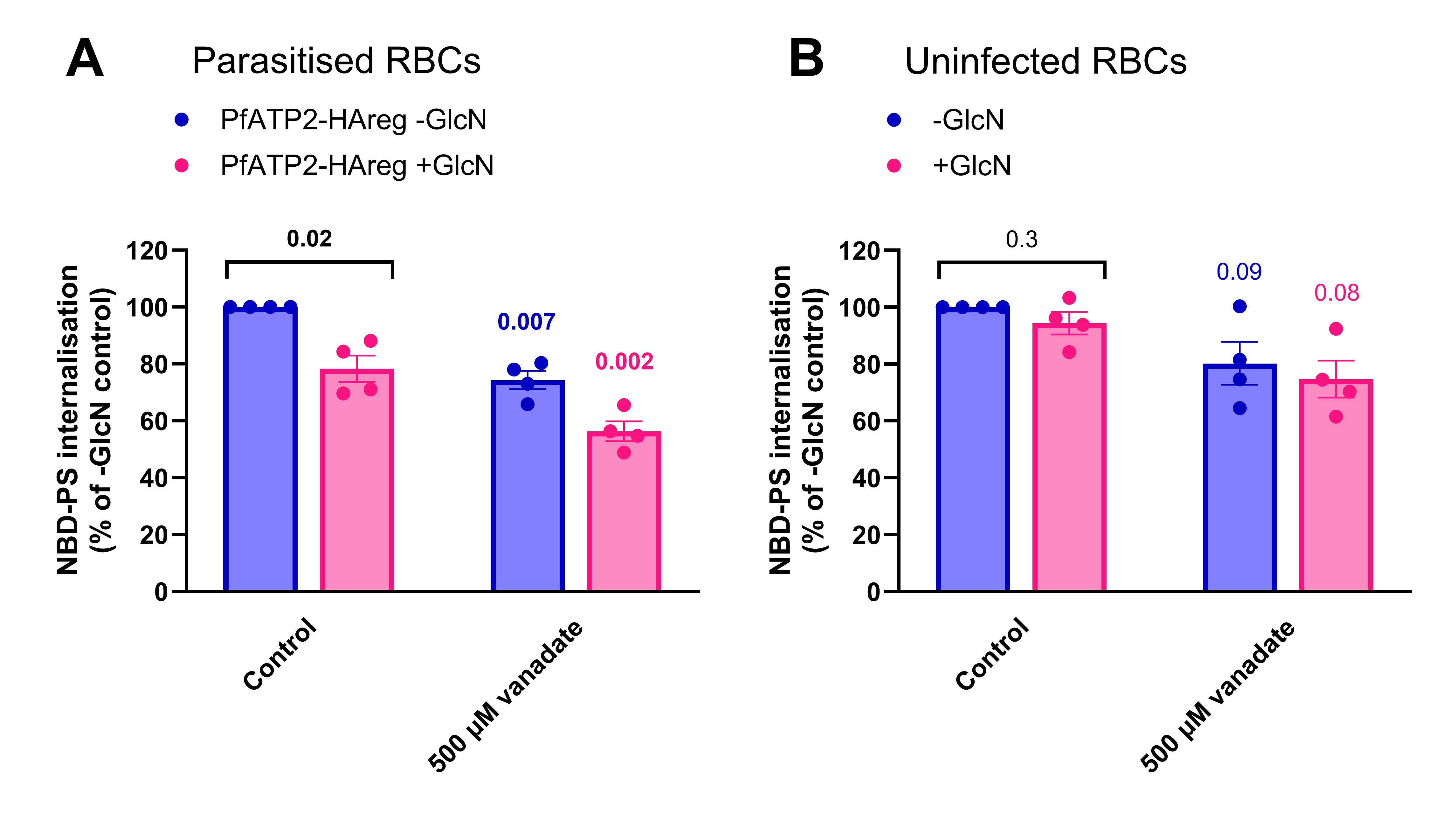

Supplement: S9 Fig — NBD-PS uptake was measured over 30 min in cells suspended in bicarbonate-free medium at 37˚C. The cells were either exposed to 5 mM GlcN for two days in the lead up to the experiment to reduce PfATP2-HA expression (+GlcN; pink) or not exposed to GlcN (-GlcN; blue). GlcN was not present during the NBD-PS uptake measurements. The bars and error bars show the mean ± SEM (from four independent experiments performed on different days, with all conditions tested concurrently) and the symbols show the data from individual experiments. The data are expressed as a percentage of the NBD-PS fluorescence (geometric mean) measured in the -GlcN Control. The NBD-PS fluorescence for the -GlcN Control (geometric mean) was higher in parasitised erythrocytes (ranging from 2406 in experiment 1 (lowest) to 52323 in experiment 3 (highest)) than in uninfected erythrocytes (ranging from 795 in experiment 1–9143 in experiment 3). Statistical comparisons were made using ratio paired t-tests on the pre-normalised data (NBD-PS fluorescence (geometric mean)). The P values are shown, with values indicating statistical significance (P < 0.05) shown in bold. Coloured values are the P values for comparisons with the solvent control for the same cell type. (TIF) [file ppat.1013645.s010.tif]

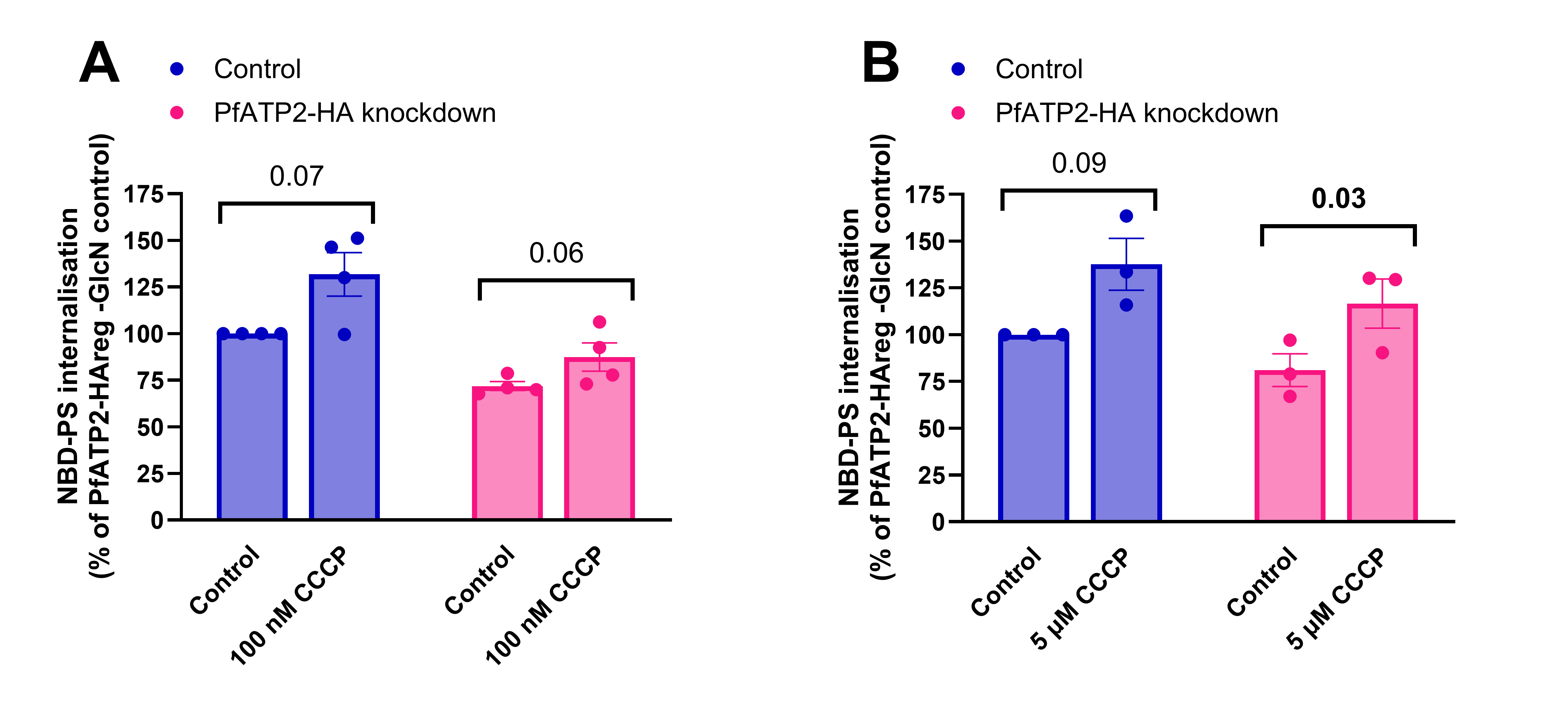

Supplement: S10 Fig — NBD-PS internalisation by isolated trophozoite-stage parasites was measured at 15˚C over 9 min. Symbols show data from each individual experiment with Control parasites (PfATP2-HAreg -GlcN; blue) and PfATP2-HA knockdown parasites (exposed to 5 mM GlcN for two days; pink). GlcN was not present during the NBD-PS uptake measurements. The bars and error bars show the mean ± SEM from four (A) or three (B) independent experiments (each performed on different days). The data are expressed as a percentage of the NBD-PS fluorescence (geometric mean) observed in Control parasites that were not treated with CCCP (0.1% v/v DMSO; solvent control). The P values from ratio paired t-tests performed with the pre-normalised data are shown (with significant values (P < 0.05) shown in bold). (TIF) [file ppat.1013645.s011.tif]

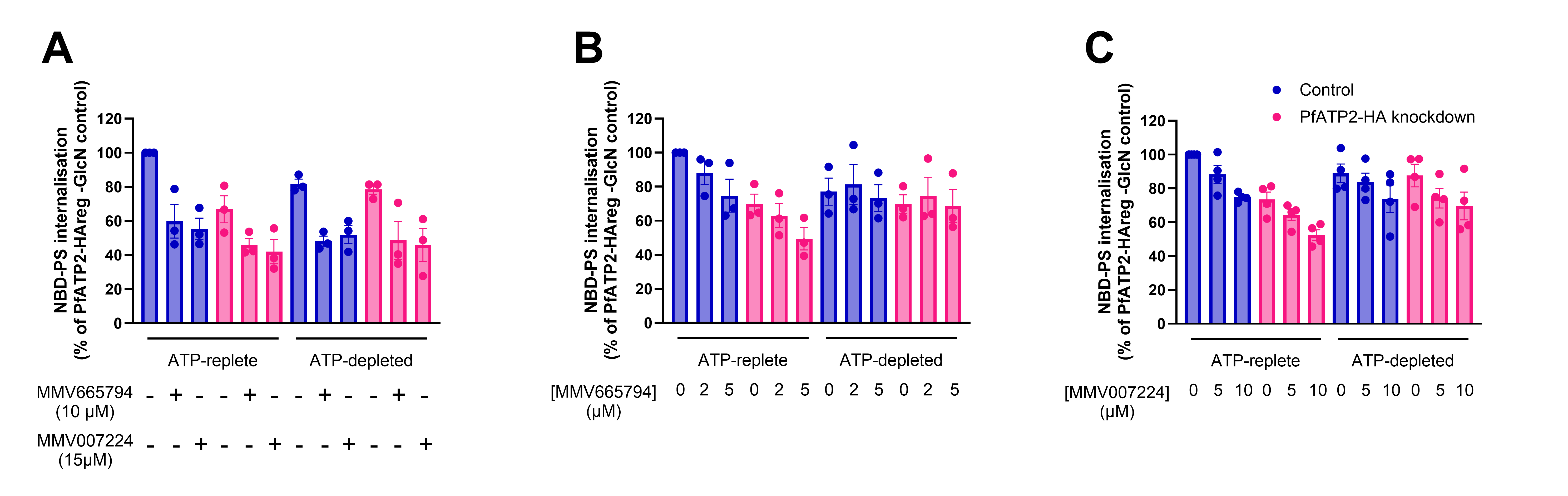

Supplement: S11 Fig — NBD-PS internalisation was measured over 9 min in isolated trophozoite-stage parasites (ATP-replete parasites suspended in pH 7.1 Physiological Saline or ATP-depleted parasites suspended in Glucose-free Saline) at 15˚C, in the absence (0.1% v/v DMSO; solvent control) or presence of the compounds and concentrations indicated. The experiments were performed with PfATP2-HA knockdown parasites (pink; from cultures exposed to 5 mM GlcN for two days) and Control parasites (blue; PfATP2-HAreg -GlcN). GlcN was not present when parasites were exposed to NBD-PS. The data are expressed as a percentage of the NBD-PS fluorescence (geometric mean) measured in the ATP-replete parasites that were not exposed to GlcN or test compound. The data are from three (A,B) or four (C) independent experiments, in which all the conditions/parasite types shown within one panel were tested concurrently. The symbols show the data from individual experiments; the bars and error bars show the mean ± SEM. The solvent control data in A are the same as three of those shown in Fig 5E (as vanadate and the MMV compounds were tested together in three experiments). The data shown in the Fig 6 insets were derived from the same experiments as the data shown in this Figure (but in the case of the Fig 6 insets were normalised to the data for ATP-depleted parasites that were not exposed to GlcN or test compound). (TIF) [file ppat.1013645.s012.tif]

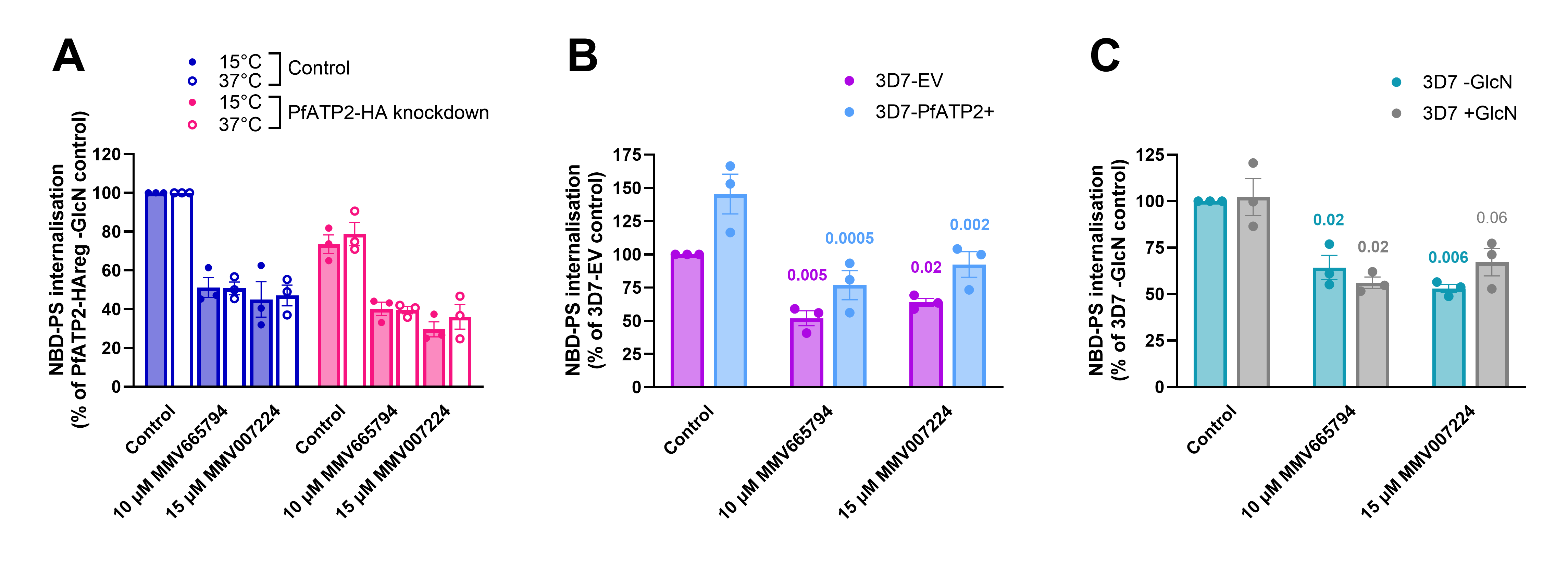

Supplement: S12 Fig — (A) PfATP2-HA knockdown (pink; from cultures exposed to 5 mM GlcN for two days) and Control (blue; PfATP2-HAreg -GlcN) parasites were suspended in pH 7.1 Physiological Saline at either 37˚C or 15˚C, in the absence (solvent control) or presence of 10 µM MMV665794 or 15 µM MMV007224, with NBD-PS internalisation measured at the 9 min time point. As noted in S8B Fig, the NBD-PS fluorescence (geometric mean) was higher at 37˚C than at 15˚C. A three-way ANOVA performed with the pre-normalised data revealed a significant effect of the MMV compounds (P = 0.003), temperature (P = 0.004) and PfATP2-HA knockdown (P = 0.05) on NBD-PS uptake, but no significant effect of temperature on the effects of the MMV compounds (P = 0.07) or PfATP2-HA knockdown (P = 0.7). (B,C) NBD-PS uptake (9 min, 15°C) in 3D7-PfATP2+ (light blue) and 3D7-EV (purple) parasites (B) and 3D7 parasites (+GlcN (grey) or -GlcN (teal); C) in the absence (solvent control) or presence of 10 µM MMV665794 or 15 µM MMV007224. The P values for comparisons with the solvent control for the same parasite type, from lognormal one-way ANOVAs with post hoc Dunnett’s tests performed on the pre-normalised data, are shown (with significant values (P < 0.05) indicated in bold). In all panels, the data are from three independent experiments (performed on different days; with all conditions tested concurrently) and are expressed as a percentage of the NBD-PS fluorescence (geometric mean) measured under the conditions indicated on the y axis. The symbols show the data from individual experiments, and the bars and error bars show the mean ± SEM. GlcN was not present during the measurements. The MMV compounds were tested at the same time as vanadate; thus the control data shown in panels A, B and C are the same as those shown in Figs 5A (inset), S8B and 5D (inset), respectively. (TIF) [file ppat.1013645.s013.tif]

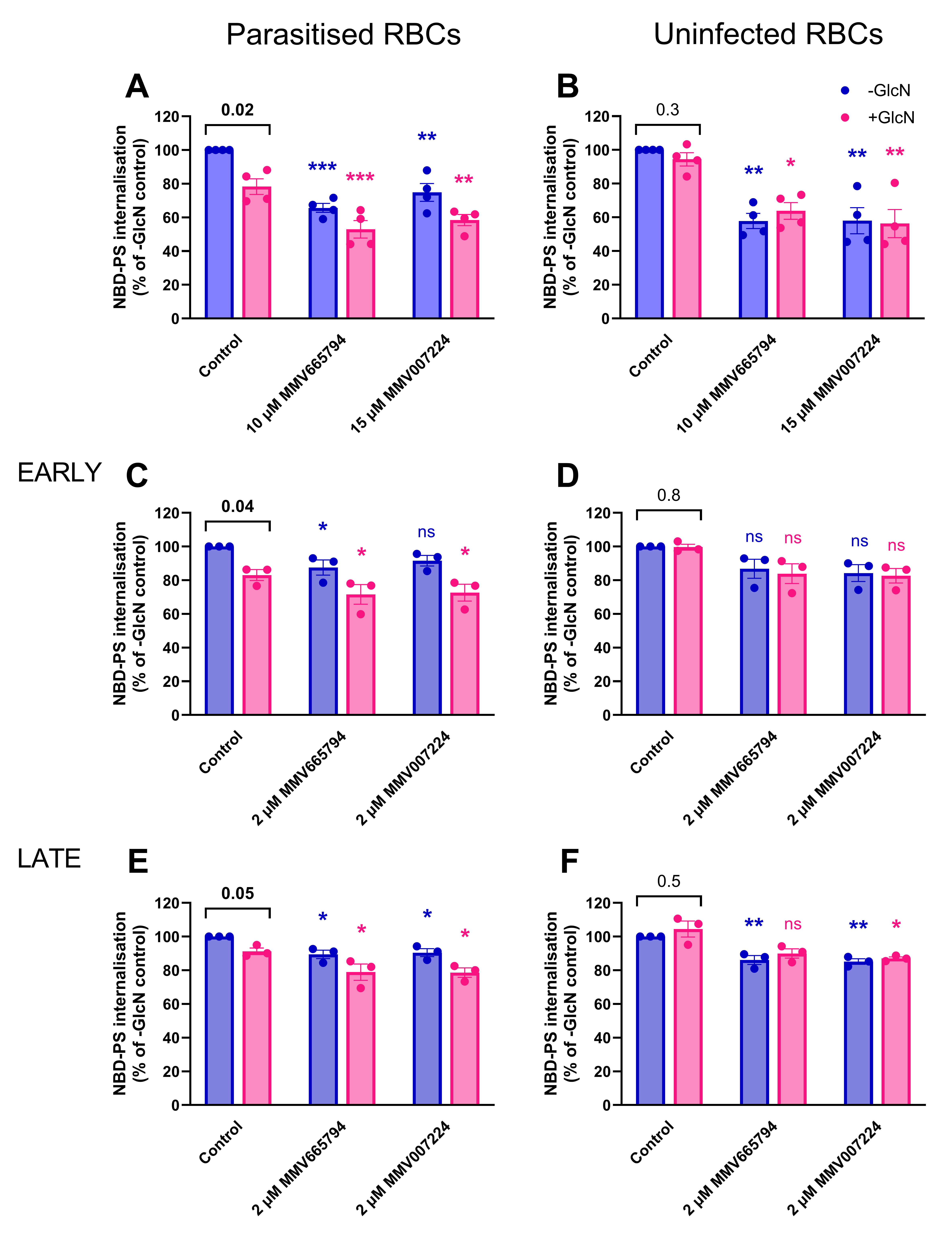

Supplement: S13 Fig — NBD-PS uptake was measured over 30 min in cells suspended in bicarbonate-free medium at 37˚C. The cells were either exposed to 5 mM GlcN for two days in the lead up to the experiment to reduce PfATP2-HA expression (+GlcN; pink) or not exposed to GlcN (-GlcN; blue). GlcN was not present during the NBD-PS uptake measurements. The bars and error bars show the mean ± SEM (from four (A,B) or three (C-F) independent experiments performed on different days (with all conditions tested concurrently) and the symbols show the data from individual experiments. The data are expressed as a percentage of the NBD-PS fluorescence (geometric mean) measured in the -GlcN Control. The NBD-PS fluorescence for the -GlcN Control (geometric mean) was higher in parasitised erythrocytes (ranging from 2406 (lowest) to 52323 (highest)) than in uninfected erythrocytes (ranging from 795 (lowest) to 9646 (highest)). In A and B, the MMV compounds were tested in the same experiments as vanadate; thus, the Control data are the same as those shown in S9 Fig. The experiments for which data are shown in C and D were performed 5.0-5.5 h earlier than those for which data are shown in E and F. In two of the three experiments, parasites were isolated from a sample of the cultures (-GlcN and +GlcN); for the later measurements (E), the mean parasite volumes had increased to 130–138% of those measured at the earlier timepoint (C). The P values in black are the results of ratio paired t-tests performed with the pre-normalised data (NBD-PS fluorescence (geometric mean)) (with bold indicating statistical significance (P < 0.05)). The data obtained for each cell type in the presence of MMV compounds was compared to the solvent control data using lognormal one-way ANOVAs with post hoc Dunnett’s tests; ns, not significant (P > 0.5), *P < 0.05, **P < 0.01, ***P < 0.001. (TIF) [file ppat.1013645.s014.tif]

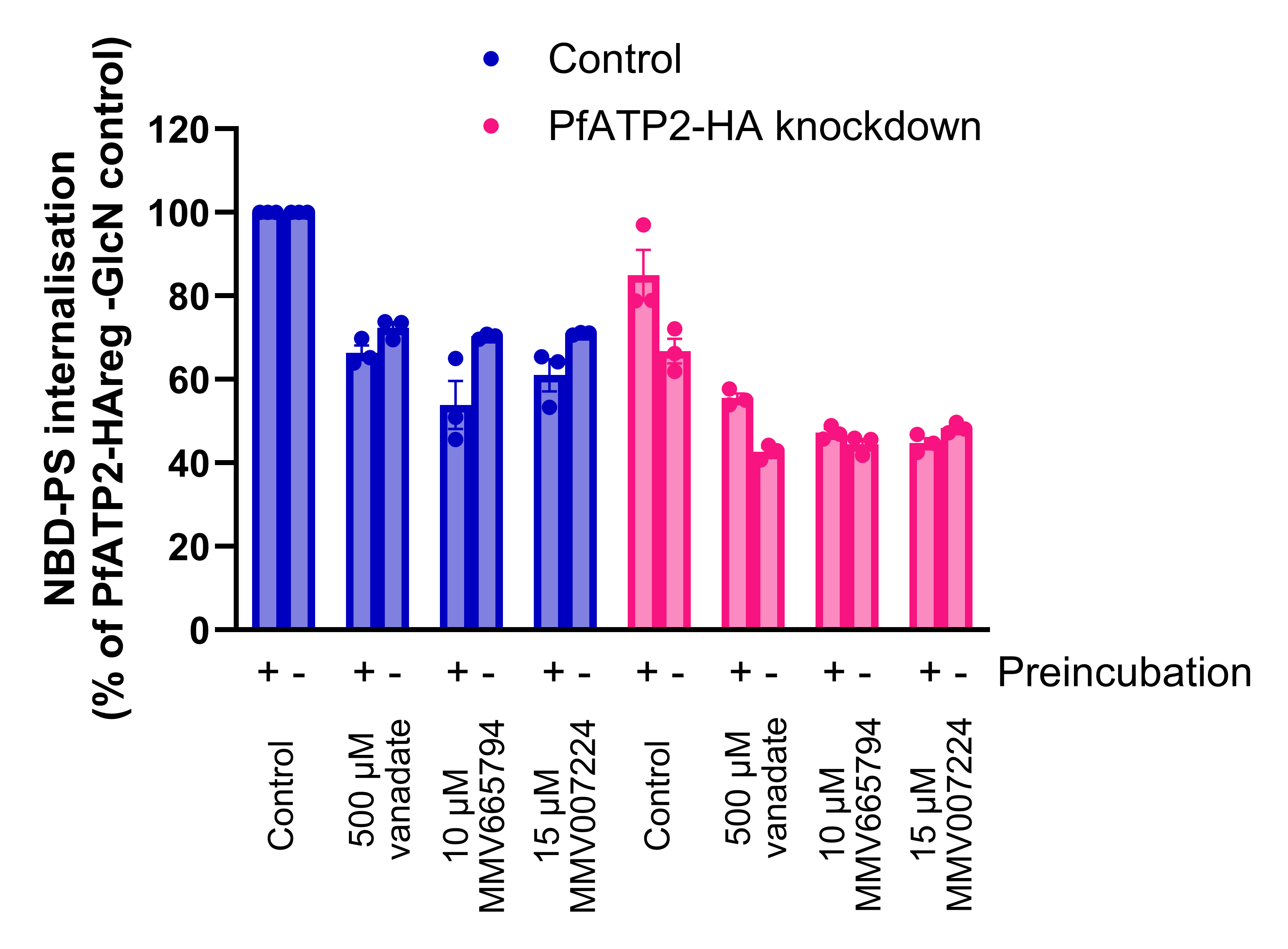

Supplement: S14 Fig — NBD-PS internalisation was measured over 9 min at 15˚C in isolated trophozoite-stage parasites suspended in pH 7.1 Physiological Saline. The cells were either exposed to 5 mM GlcN for two days in the lead up to the experiment to reduce PfATP2-HA expression (+GlcN; pink) or not exposed to GlcN (-GlcN; blue). GlcN was not present during the measurements. The bars and error bars show the mean ± SEM (from three independent experiments performed on different days, with all conditions tested concurrently) and the symbols show the data from individual experiments. The data are expressed as a percentage of the NBD-PS fluorescence (geometric mean) measured in the -GlcN Control. To determine whether preincubation had a significant effect on the activity of any of the compounds, two-way ANOVAs were performed for each compound using the normalised data. Preincubation did not significantly affect the activity of vanadate (P = 0.2), MMV665794 (P = 0.09) or MMV007224 (P = 0.3). In two experiments, 5 µM CCCP was tested alongside other compounds for which data are shown in this Figure. Thus, the Control data for two of the experiments in this Figure (preincubation condition) are the same as those shown in S10 Fig. (TIF) [file ppat.1013645.s015.tif]

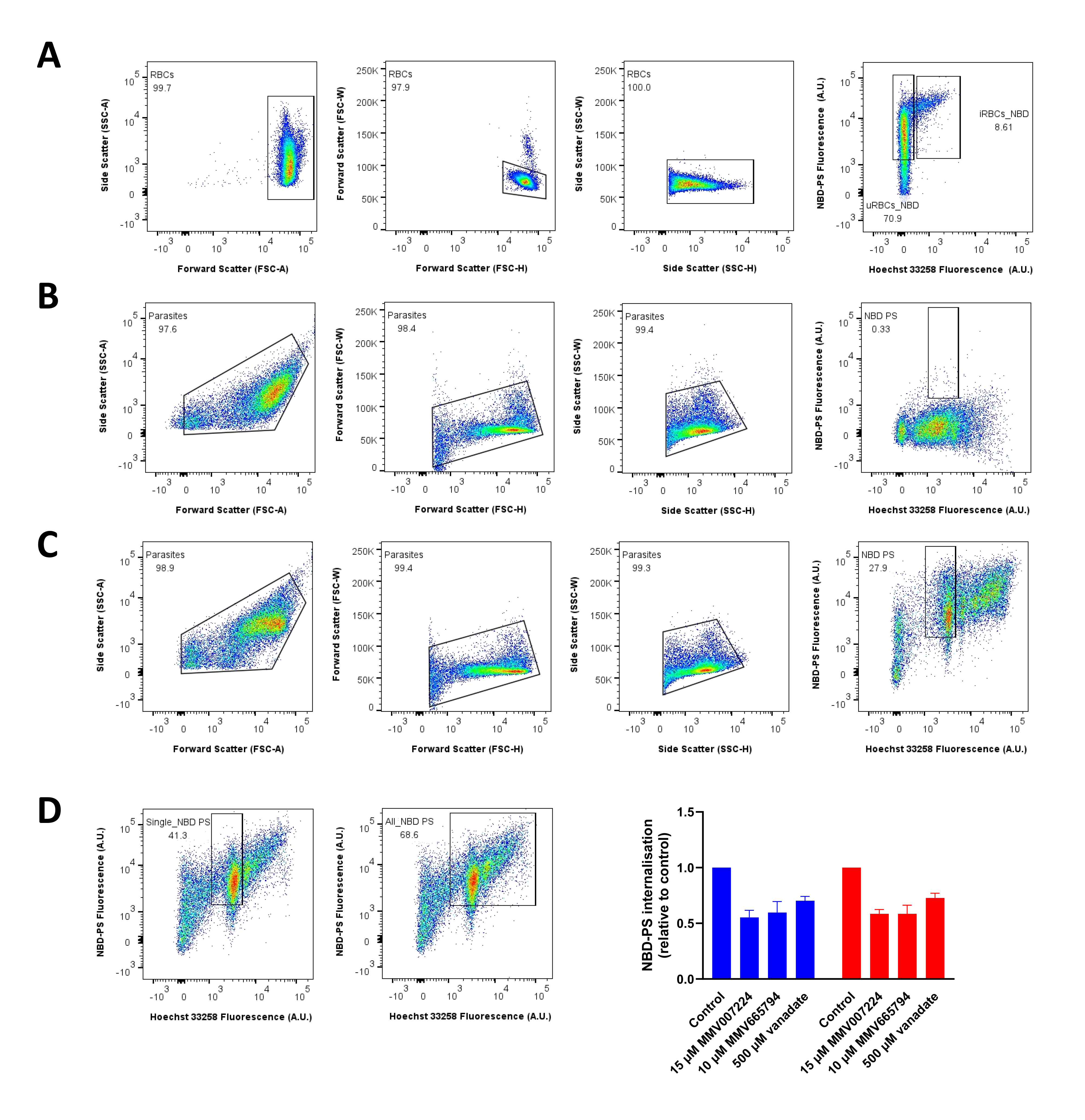

Supplement: S15 Fig — RBCs (A; uninfected and infected with P. falciparum) or isolated parasites (PfATP2-HAreg -GlcN; B-D) were first gated in a plot of SSC‐A versus FSC-A to exclude debris. Cells were then gated in plots of FSC‐W versus FSC‐H and SSC‐W versus SSC‐H, in order to exclude doublets and aggregates. The cells were then gated in a plot of Hoechst 33258 fluorescence and NBD-PS fluorescence. The data in each panel are from a single experiment, representative of three or more independent experiments. (A) Gating strategy for uRBCs and iRBCs (infected with PfATP2-HAreg parasites) (-GlcN, 0.1% v/v DMSO condition). (B-C) Gating strategy for isolated trophozoite-stage parasites (PfATP2-HAreg -GlcN) that were not exposed to NBD-PS (B) or that were exposed to NBD-PS for 9 min at 15˚ (C). Except for the NBD-PE experiment for which data are shown in S4 Fig (for which a similar strategy to that shown for ‘All NBD PS’ in D was used), the gating strategy shown in panel C was used throughout this study. However, forming a larger gate encompassing parasites with varying Hoechst 33258 fluorescence levels (which are expected to be more diverse in level of maturity, with some parasites likely having multiple nuclei) yielded similar results (D). In the bar graph, the results obtained using the smaller gate shown on the left (‘Single_NBD PS’) are shown in blue, and those obtained using the larger gate shown on the right (‘All_NBD PS’) are shown in red. The data are for ATP-replete PfATP2-HAreg Control parasites (-GlcN) that were exposed to DMSO (0.1%; solvent control), 15 µM MMV007224, 10 µM MMV665794 or 500 µM vanadate (data from the same experiments are also shown in Figs 5E and S11A). The bars and error bars show the mean + SEM from three independent experiments. (TIF) [file ppat.1013645.s016.tif]
